# Gastrointestinal endoscopic image classification using a hybrid modified inception network and a customized vision transformer with tree growth feature selection

**DOI:** 10.1038/s41598-026-55235-z

**Published:** 2026-06-23

**Authors:** Rehana Rauf, Samia Ijaz, Saima Gulzar Ahmad, Ehsan Ullah Munir, Naeem Ramzan

**Affiliations:** 1 Department of Computer Science, HITEC University Taxila, Taxila, Pakistan; 2 Department of Computer Science, COMSATS University Islamabad, Wah Campus, Islamabad, Pakistan; 3 School of Computing, Engineering and Physical Sciences, University of the West of Scotland, High Street, Paisley, PA1 2BE UK

**Keywords:** Image classification, Inception, Colorectal, Colonoscopy, Endoscopy, Gastrointestinal, Vision transformer, Kvasir, Tree growth algorithm, Cancer, Computational biology and bioinformatics, Diseases, Gastroenterology

## Abstract

The Gastrointestinal (GI) tract plays a vital role in digestion by breaking down food into essential nutrients. Disorders such as bleeding lesions, ulcerative colitis, Inflammatory Bowel Disease (IBD), constipation, diarrhea, abdominal pain, nausea, and vomiting may indicate serious chronic conditions, including cancer. GI malignancies are among the leading causes of cancer-related mortality worldwide; however, early and accurate diagnosis can significantly reduce fatality rates. Existing endoscopic AI systems often struggle to generalize across datasets. They also face challenges in capturing both local lesion characteristics and long-range contextual information. To address this, the proposed study explores deep learning-based methods for automated classification of gastrointestinal diseases using endoscopic images. Two models were developed: a modified Inception architecture with four blocks and a customized Vision Transformer (ViT-3) consisting of three transformer encoder blocks. Deep features were extracted from both models, which were fused using a weighted fusion strategy. The redundant features were reduced using the Tree Growth Algorithm (TGA). The optimized feature set was then classified using machine learning classifiers to improve diagnostic performance. To evaluate its effectiveness, the framework was tested on two widely used benchmark datasets, Kvasir v1 and Kvasir v2, which include 4,000 and 8,000 images, respectively across eight GI disease categories. On the Kvasir v1 dataset, the proposed method achieved an accuracy of 98.9%, along with sensitivity, precision, and F1-score values of 98.87%, 98.86%, and 98.86%, respectively. Similar performance was observed on the Kvasir v2 dataset. These findings demonstrate that the proposed method provides a reliable and effective solution for early identification and classification of gastrointestinal diseases from endoscopic images.

## Introduction

Several kinds of disorders can affect the human digestive system. Three of the most prevalent cancers among the eight cancers that develop in the Gastrointestinal (GI) tract are esophageal, stomach, and colorectal cancers^1^. In addition, GI disorders are frequent digestive issues, including conditions like gastric ulcers, colon polyps, and GI tumors^2^. GI disorders, such as colorectal cancer, Inflammatory Bowel Disease (IBD), and gastric ulcers, are significant global health issues affecting millions of people annually. GI tract problems are significantly prevalent in both developed and underdeveloped countries^3^. For instance, it has been reported that there are around 27,510 new cases of gastrointestinal disorders recorded each year in the United States alone, with 11,140 deaths^4^. In 2021, GI healthcare spending was $111.8 billion. A GI diagnosis or symptom led to 14.5 million ER visits and 2.9 million hospital admissions. There were 315,065 new GI cancers diagnoses leading to 281,413 fatalities^5^.

The widespread prevalence of gastrointestinal tract disorders (GI) and the high death rate associated with them necessitate an urgent need for accurate diagnosis^6^. Accurate and timely detection of these diseases is necessary for better treatment and improved patient survival rates^7^. In practice, gastrointestinal diseases often present with subtle or overlapping features. This not only complicates the diagnosis, but the time needed for evaluation is also increased. In addition, reliance on expert interpretation may cause further delays. Consequently, clinical decision-making and overall patient outcomes are impacted.

GI disorders are usually diagnosed with the help of diagnostic tests such as endoscopy, X-ray and CT scans. Among these, the gold standard for investigating the GI system is endoscopic examinations.

These imaging techniques produce considerable amounts of data that need to be interpreted, which can increase the time required for diagnosis and also raise the likelihood of human error. Therefore, there is an increasing demand for automated and computer-assisted methods to improve the efficiency and reliability of diagnosis.

Numerous studies have been conducted in the field of diagnostic imaging by using deep learning (DL) and computer vision, e.g., magnetic resonance imaging (MRI) and computed tomography (CT)^8^, capsule endoscopy^9^, and X-ray^10^. Recent advances in DL have shown great promise in automating medical image analysis and improving diagnostic accuracy.

However, a significant gap in the literature still exists. Most of the existing methods adopt single model architectures to capture either local or global features but not both at the same time. The issue of feature redundancy in deep feature spaces remains largely unexplored. The integration of CNN and ViT architectures with biologically inspired techniques for choosing features is also under-explored for GI endoscopic image classification. Existing studies also struggle with several critical issues such as CNNs’ incapability to model long-range dependencies, the significant data and computational demands of transformers, lack of specialized feature selection procedures, and limited cross-dataset validation which raises generalizability concerns.

Despite the promising results shown by the recent studies, several limitations still affect GI endoscopic image classification approaches. First, many existing methods are based on single-model architectures, which may not be able to effectively capture both local and global features. Second, there is insufficient consideration of feature redundancy and selection, which can adversely affect classification accuracy and computational efficiency. Third, ensemble strategies that combine CNNs and ViTs in GI endoscopic image classification are still under-explored, especially in combination with biologically inspired feature selection methods.

The mentioned gaps serve as motivation for the development of a powerful hybrid framework that leverages the advantages of both architectures while overcoming the challenges of feature optimization. To address these issues, in this study we propose an ensemble-based GI endoscopic image classification system, where a modified Inception-4-block model is used for better local feature extraction and a customized ViT is designed for global contextual modelling. In addition, a Tree Growth Algorithm (TGA) feature selection method is used to find the most discriminating features, remove redundancy and improve classification performance. The primary aim of this study is to develop a robust hybrid framework that simultaneously captures local and global features. The proposed method is also able to remove redundant information to achieve accurate and efficient GI disease classification on the Kvasir v1 and Kvasir v2 benchmark datasets. The main contributions of this work are summarized as follows:

- We designed an Inception architecture with four blocks. Each block utilizes multiple convolutional layers to improve deep feature extraction.
- We implemented a weighted fusion technique. Here, the feature vectors from the Inception-4 architecture and the ViT-3 Transformer were combined to leverage the strengths of both architectures.
- To refine the fused feature set, we employed TGA. This approach selects the most prominent and essential features resulting in improved classification accuracy.

The remainder of this article is organized as follows: Sect.  2 examines the related research work, Sect.  3 provides an in-depth overview of the proposed methodology, experimental results and performance evaluation are provided in Sect.  4 and Sect.  5 concludes the work.

## Literature review

This section reviews recent studies on GI endoscopic image classification using machine learning and deep learning methods, highlighting their key contributions and limitations. The reviewed works cover a range of approaches, including CNN-based architectures, Vision Transformer models, transfer learning techniques, and hybrid ensemble methods. The limitations identified across these works collectively motivate the development of the proposed framework presented in this study.

Convolutional Neural Network (CNN) models, including VGGNet^11^ and ResNet^12^, have demonstrated strong feature extraction capabilities by efficiently acquiring local features in medical images. These models have been widely used for GI endoscopy tasks such as polyp identification, lesion classification, and disease diagnosis, attaining performance similar to that of trained experts in some studies^13,14^. In addition, Vision Transformers (ViT) have emerged as strong alternatives by modeling long-range relationships through self-attention processes, providing greater global contextual understanding of complex visual structures^15^. Recent publications have examined ViT-based architectures for endoscopic image processing and demonstrated encouraging results, notably in handling various lesion presentations and complicated backdrops^16,17^. However, CNNs frequently struggle to capture global semantic relationships due to their limited receptive fields, while ViT typically requires large-scale annotated datasets and substantial computational resources, limiting their generalization capability in medical imaging scenarios where labeled data are scarce^18^.

Over the past two decades, medical imaging has made significant progress in diagnosing disorders across several human organs^19,20^. Recent research in endoscopy^21^, histopathology^22^, speech recognition^23,24^, rheumatology^25^, automatic captioning^26^, fundus imaging^27^, and telemedicine^28^ has applied machine learning (ML) and computer vision algorithms. Recently, ViTs have achieved state-of-the-art performance in a range of digital AI-based health applications. There is growing interest in using DL approaches to medical image classification, particularly in GI imaging^29^. Methods ranging from traditional CNNs^30^ to more sophisticated hybrid models, have been explored to address GI disease diagnosis such as detecting tumors, polyps, and other abnormalities in endoscopic, colonoscopy, and radiographic images.

In recent studies, researchers have emphasized improving classification accuracy to detect digestive system diseases as an essential goal. The current section briefly overviews some of the most current studies using ML and DL methods. Authors in^31^ introduced a hybrid approach which combined texture-based features along with deep learning approaches, obtaining 95.02% accuracy on Kvasir dataset images. However, no feature selection strategy was employed, leaving the risk of redundant features affecting classification efficiency unaddressed, and the model was not validated across multiple datasets, limiting conclusions about its generalizability. Additionally, research on initial stage diseases including gastrointestinal cancer reveals the significance of CNN-based frameworks for precise identification. Yoshiok et al.^32^ performed a comparative study of CNN architectures, demonstrating higher F1-scores for GoogLeNet and higher prediction confidence with MobileNetV3. GoogLeNet achieved approximately 84.6% accuracy in the identification of esophagitis and z-line pictures from Kvasir dataset. The limited accuracy suggests that the single-model CNN architectures are not able to capture the full complexity of GI endoscopic images and no ensemble or feature fusion strategy was investigated to further improve performance.

In^33^, an improved LSTM-based CNN was proposed for the classification of gastrointestinal system. They used LSTM to enhance CNN performance and obtained improved outcomes than the CNN + ANN and CNN + SVM classifiers. However, the increased architectural complexity raises queries regarding computational efficiency, and no ablation analysis was done to verify the specific contribution of the LSTM component. A further significant contribution was done by^34^, which explored transfer learning algorithms on the Kvasir dataset, which consists of eight GI image categories. They trained many pre-trained models with data pre-processing and augmentation approaches. They conducted experiments that showed different accuracy rates, such as VGG16 (97.61%), CNN (95.22%), ResNet50 (96.61%), Xception (77.94%), and DenseNet121 (88.11%). Their proposed Efficient-B1 model is the best among these with the accuracy of 99.22%. The accuracy was impressive, but it was not validated across different datasets, raising concerns of overfitting to the Kvasir dataset and limiting conclusions about real-world clinical generalizability.

Authors in^35^ performed a comprehensive classification on the HyperKvasir dataset with multiple deep learning approaches to diagnose gastrointestinal disorders. Their work evaluated several pre-trained CNN architectures, including ResNet50, InceptionV3, DenseNet201, EfficientNet-B0, VGG16, and MobileNetV2. The experimental results indicated that EfficientNet-B0 achieved the best performance with the highest accuracy of 96.25%. However, the study was based on single-model classification without exploring the feature fusion or feature selection strategies, which may leave the redundant information in the high dimensional feature space. A recent study^36^ has investigated hybrid models for interpretation and analysis of endoscopic images with a focus on proactive diagnosis of gastrointestinal diseases. They used pre-trained models such as GoogLeNet (88.2% classification accuracy), MobileNet (86.4% classification accuracy) and DenseNet121 (85.3% classification accuracy). Hybrid techniques of CNNs with FFNN (Feedforward Neural Networks) and XGBoost further improved the performance with a maximum accuracy of 97.25%. The dependency of GVF-based segmentation as a preprocessing step introduces extra computational burden and any segmentation inaccuracies might propagate imperfections into the classification pipeline. These models merged fused CNN features with gradient vector flow (GVF)-based segmentation algorithms demonstrating how hybrid techniques can improve diagnostic accuracy.

A comparative study^37^ was conducted to assess the performance of several deep learning models for classification of gastrointestinal diseases based on wireless capsule endoscopy images. The study investigated popular CNN architectures like AlexNet, ResNet18, VGG16, InceptionV3 and GoogLeNet. The dataset consists of wireless capsule endoscopy images of different categories of gastrointestinal diseases. According to the evaluation results, GoogLeNet achieved the best performance with an F1-score of 98.1% and an accuracy of 98.4%. The study was based only on single-model evaluation and did not explore feature fusion across architectures. The study was limited to wireless capsule endoscopy images which are quite distinct from conventional colonoscopy images.

In^31^, the authors have employed pre-trained DenseNet-201, ResNet-18, and VGG-16 CNN feature extractor models. Adding a global average pooling (GAP) layer to create deep features achieved an accuracy of 97.38%. The proposed method revealed 8 different groups of digestive tract disorders. However, there is no feature selection strategy applied, which leaves the risk of redundant features that affect classification efficiency unaddressed. Additionally, the model was not validated in multiple datasets, which limits the generalizability of the conclusions that can be drawn. Similarly, in^32^, GI disorders are classified using VGG-16, ResNet-18 and GoogLeNet models and VGG-16 achieves an accuracy of 96.33%.

In^33^, researchers have suggested an innovative method for diagnosing gastrointestinal disorders utilizing computer-aided diagnosis (CADx) techniques, with the objective of enhancing disease detection through deep learning (DL) methodologies and sophisticated image processing. The research indicated that a subspace discriminant classifier utilized on the Kvasir dataset attained a classification accuracy of 95.0%. Furthermore, transfer learning methodologies have been thoroughly examined. In^34^, a new method based on transfer learning and using different pre-trained weights was tested on Kvasir dataset. The best model was EfficientNetB0, which had an accuracy of 98.01% and a recall and precision of 98%.

Another study^35^ employs a CNN-based method to detect the difference between patients with ulcerative colitis and those without it, showing how severe the disease is in endoscopic images. Furthermore, new frameworks have been proposed in^36^ for angiectasia detection using Generative Adversarial Networks (GANs) in wireless capsule endoscopy. The proposed approach achieved a sensitivity of 88% and a specificity of 99.9% at the pixel-wise localization level, whereas it reached a sensitivity of 98% at the frame-wise detection level. A recent study has developed a deep ensemble neural network for effective abnormality diagnostic in the GI tract.

Recent advancements in deep learning have considerably improved gastrointestinal (GI) image classification. DeepGI has utilized convolutional neural networks (VGG16, MobileNetV2, and Xception) on a GI dataset of approximately 4,000 images, achieving an accuracy of 96.5%^38^. The accuracy achieved is considerably lower than state-of-the-art ensemble approaches, suggesting that prioritizing interpretability without optimizing the classification architecture is insufficient for practical clinical deployment. Similarly, GastroViT has employed a ViT ensemble using MobileViT variants, along with Grad-CAM and SHAP for interpretability, on Hyper-Kvasir dataset, achieving accuracy of 92%^39^. The relatively modest accuracy suggests that transformer-based models require larger annotated datasets to fully realize their potential, and no hybrid CNN-ViT architecture was explored to leverage the complementary strengths of both paradigms.

Ensemble and feature-fusion approaches have further enhanced performance. A weighted ensemble combining NasNet-Mobile and EfficientNet, trained on Hyper-Kvasir and evaluated on Kvasir v1 and v2, has reported accuracies of 97.83% and 98.45%, respectively^40^. The high computational cost of combining two large pre-trained networks raises concerns about deployment in resource-constrained clinical environments, and no feature selection mechanism was incorporated to reduce feature space dimensionality. In addition, a fusion method integrating modified ResNet18 and ResNet50 features with a Newton–Raphson-controlled Marine Predator Optimization algorithm has achieved 97.8% accuracy on Kvasir v2 and 98.4% on Hyper-Kvasir^41^. The computational complexity of the Marine Predator Optimization algorithm may pose challenges for real-time clinical applications, and evaluation on a single dataset limits generalizability conclusions.

Researchers in^42^ have created a DL-based approach to identify ulcers and categorize GI diseases. Using a personalized mask to segment ulcers, a recurrent CNN was employed. After feature selection, MSVM is used to classify the features, achieving an accuracy of 99.13%. However, the dependency on a personalized segmentation mask introduces additional complexity and may not generalize well across different imaging conditions and datasets. In^43^, researchers have suggested a fully automated method for identifying stomach infections using fusion, multi-type feature extraction, and rigorous feature selection. CNN features based on VGG16, discrete cosine transform, and discrete wavelet transform were extracted during the process of feature extraction, and the method achieved an accuracy of 96.5%. The absence of a dedicated feature optimization stage means that fused feature representations may contain redundant information, potentially limiting both classification accuracy and computational efficiency. The authors in^44^ proposed a deep learning model (SNet) to enhance the detection of gastrointestinal disease by addressing the challenges in endoscopic image classification. It attained 98.45% accuracy on Kvasir v1 dataset and 97.83% on the Kvasir v2. In^45^, the authors have presented a Gabor capsule network for identifying complex visual patterns on Kvasir dataset, achieving an overall accuracy of 91.50%. However, the capsule-based framework is known to suffer from high computational complexity and limited scalability to large datasets, and alternative optimization strategies were not compared. In^46^, a CNN-based wavelet transform method is suggested for GI tract disorder classification, achieving an average accuracy of 93.65% across the eight classes.

GastroNet^38^ was proposed as a framework for the detection and for classification of stomach cancers, achieving a mean accuracy of 97.3% on the Hyper-Kvasir dataset. However, the evaluation was restricted to a single dataset, limiting conclusions about the robustness and generalizability of the framework across varied GI imaging conditions.

A federated ViT framework with Clinical-Entropy Guided Quantum-Inspired Token Pruning^39^ was evaluated on curated upper- and lower-GI tract subsets of the HyperKvasir dataset under realistic non-IID federated conditions, achieving 92.33% and 90.19% micro-averaged accuracy for lower- and upper-GI classification respectively. However, the modest accuracy compared to centralized approaches highlights the performance trade-off introduced by federated training, and the complexity of quantum-inspired pruning raises concerns about practical clinical deployment.

The CONV-FAN-POX model^40^ achieved classification accuracy 98.8% on augmented data, demonstrating competitive performance compared to standard dense-based networks and existing state-of-the-art models. However, the reliance on augmented data raises concerns about real-world performance, and the absence of a dedicated feature selection mechanism leaves the feature space prone to redundancy.

A multi-head CNN model^41^ was proposed that achieved a classification accuracy of 96.72% using five-fold cross-validation, with potential for implementation in an end-to-end computer-aided diagnosis system to shorten evaluation time. However, the improvement margin remains limited, and the framework has not been validated across multiple benchmark datasets, restricting generalizability.

Table 1 presents a summary of some recent related studies.

**Table 1:** Performance comparison of related studies.

Year	References	Methods/models	Datasets	Accuracy (%)	Limitations
2025	^42^	Interpretable DL model	Kvasir v2	94.25	Limited generalization and weak interpretability due to small datasets.
2025	^43^	Transfer + NNMF + mRMR feature selection	Kvasir v2	97.8	Trained on small datasets which may limit robustness and generalization.
2025	^44^	Weighted ensemble: NasNet-Mobile + EfficientNet	Kvasir v1 & v2	97.83 / 98.45	High computational cost from deep ensemble architecture.
2024	^45^	Spatial-Attention ConvMixer	Kvasir v2	93.37	Tested mainly on Kvasir; limited cross-dataset robustness.
2023	^46^	Ensemble of DenseNet201, InceptionV3, ResNet50	Kvasir v2	95.00	Ensemble complexity increases processing cost and reduces generalization.
2022	^47^	Deep capsule + feature selection	Kvasir v1 & v2	98.20 / 98.02	Capsule-based framework may suffer from limited robustness and generalization.

Collectively, these works demonstrate rapid progress in applying deep learning to GI disease diagnosis. However, deep feature representations often contain redundant or non-informative information, which can reduce model generalizability and increase computational complexity. Despite much progress, many of the existing approaches rely on a single CNN-based architecture which is effective to extract local features but unable to model global contextual relationship in complex endoscopic images. ViTs are good at modelling global interactions, but they are not suitable for GI image analysis due to high computational cost, low customizability and difficulties with small medical datasets. Moreover, feature redundancy and high dimensional deep feature space are often neglected, and the existing ensemble methods lack sufficient architectural diversity to fully exploit the complementary feature representations. To overcome these limitations, this study proposes an ensemble framework that combines a modified Inception-4-block model with a customized ViT, and incorporates a TGA Feature Selection (TGFS) method.

## Proposed methodology

**Fig. 1:**
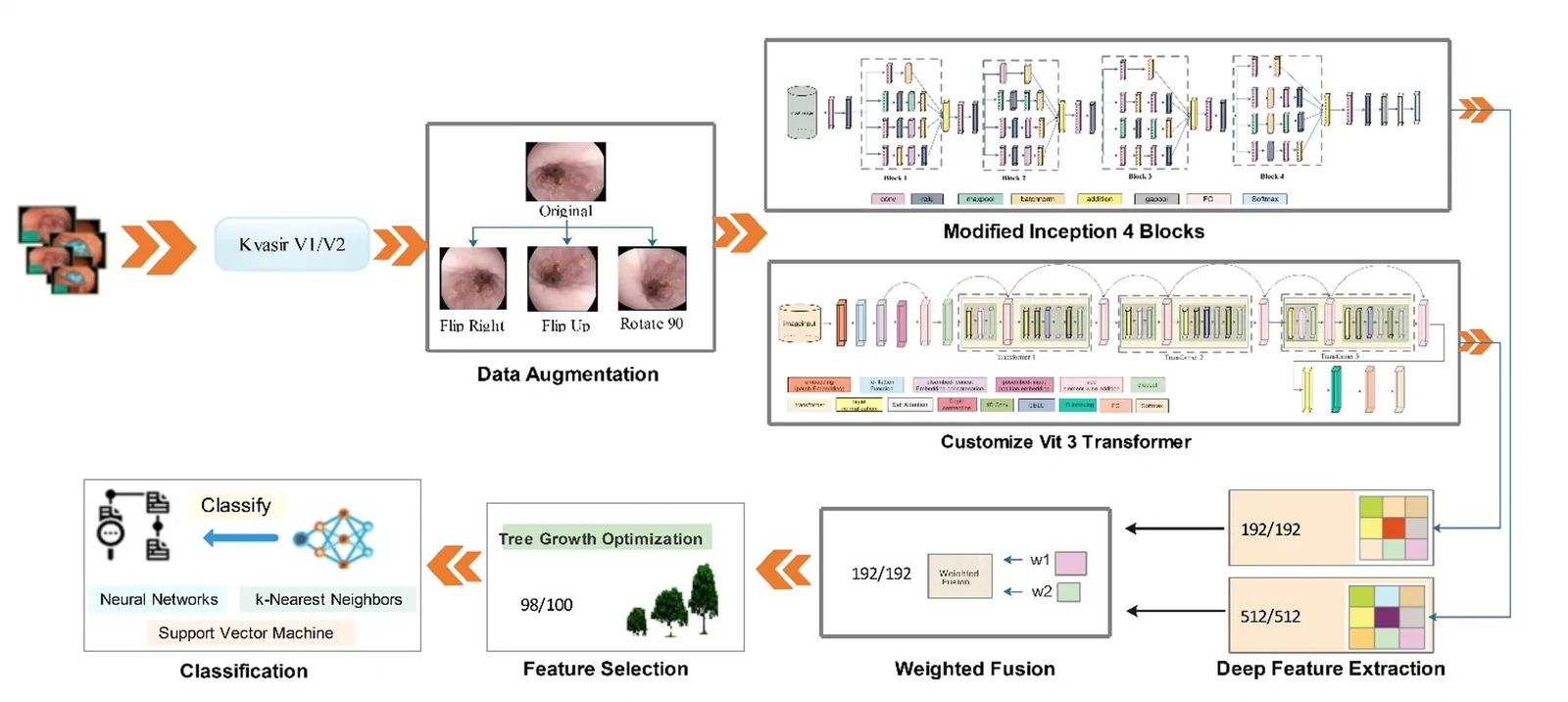
Comprehensive structure of the proposed methodology.

This section presents the proposed technique for classifying GI diseases using the Kvasir dataset^1^. The method starts with data augmentation, then trains and tests two DL models: A modified Inception-4-block model and a customized ViT-3 Transformer model. Features are extracted from both models separately and then integrated into a unified feature vector using a weighted fusion technique. Because the fused feature set may contain duplicate or non-discriminatory information, a TGA optimization method is used. This approach is chosen due to its ability to maintain solution diversity while converging reliably. In comparison to Particle Swarm Optimization (PSO) and Genetic Algorithm (GA), TGA requires fewer parameters and performs well in complex, high-dimensional feature spaces such as medical imaging applications. The optimized feature subset is then analyzed using seven classifiers to get the final categorization. Figure 1 illustrates the architecture of the suggested methodology.

### Datasets

In this work, we used two publicly available and widely employed benchmark GI endoscopy datasets, Kvasir v1 and Kvasir v2^1^. Both datasets consist of eight classes, including esophagitis, normal cecum, dyed-lifted polyps, dyed-resection margins, and polyps. The class labels are: normal-z-line, normal-pylorus, normal-cecum, esophagitis, polyps, ulcerative-colitis, dyed-lifted-polyps, and dyed-resection-margins.

**Fig. 2:**
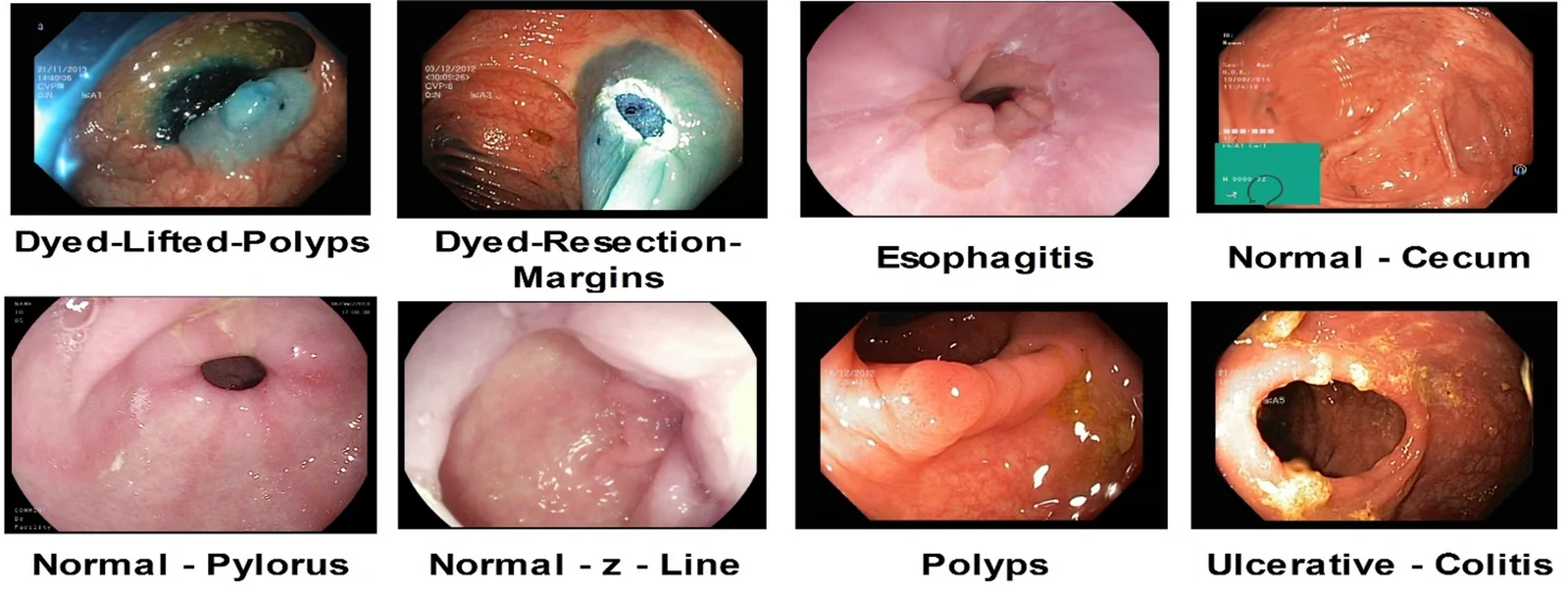
Sample images of the datasets.

In Kvasir v1, there are a total of 4000 images (each class containing 500 images). Kvasir v2 is an extended version consisting of 1000 images per class i.e. a total of 8,000 images. Both datasets reflect realistic endoscopy conditions, including changes in viewing angle, lighting, camera-to-tissue distance, and device characteristics. Because the image sizes vary, preprocessing typically involves resizing images to the required model input. Other image details are illustrated in Fig. 2.

### Data augmentation

**Fig. 3:**
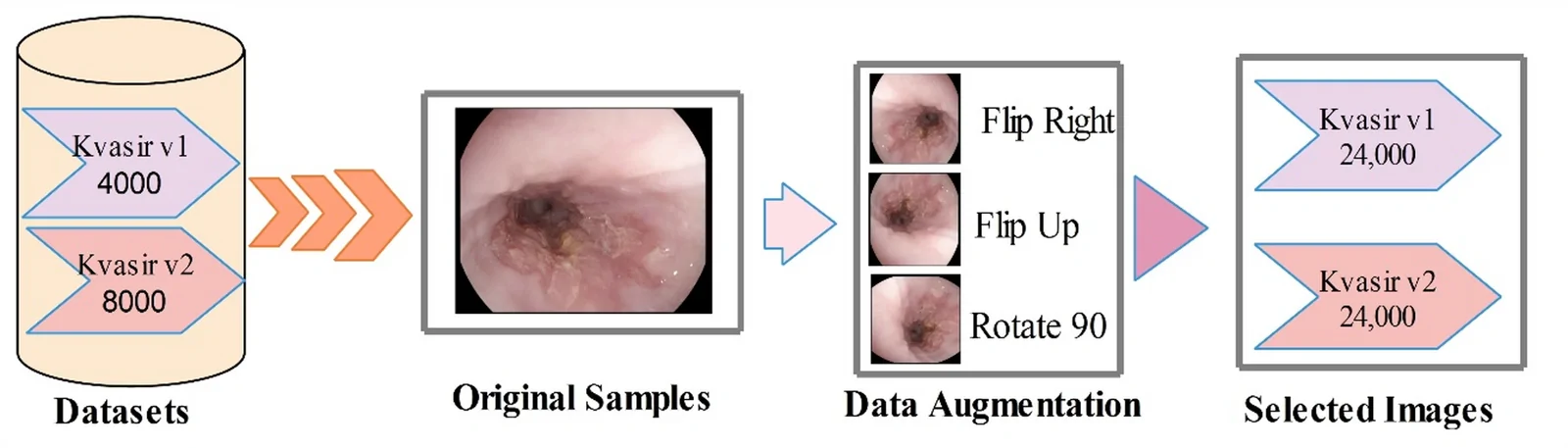
Sample images of the data augmentation.

To enable more robust learning and better generalization, data augmentation was employed to increase the volume and diversity of training and testing data. Three procedures were carried out sequentially: 90-degree rotation, vertical flipping (flip up), and horizontal flipping (flip right)^48^. From the augmented dataset, 3000 images were randomly selected. These operations simulate variations commonly observed in real-world endoscopic procedures, such as changes in orientation and viewpoint. As a result, the Kvasir v1 dataset was expanded from 4,000 to 24,000 images, while the Kvasir v2 dataset increased from 8000 to 24,000 images after augmentation. Sample augmented images are illustrated in Fig. 3, and Table 2 provides additional information on class-wise image distribution before and after augmentation.

**Table 2:** Dataset details.

Datasets	Number of classes	Number of images in each class		Total number of images	
		Original	After augmentation	Original	After augmentation
Kvasir v1	8	500	3000	4000	24,000
Kvasir v2	8	1000	3000	8000	24,000

### Modified inception-4-block model

In this study, we used a custom Inception model. The proposed architecture consists of four blocks, and each block includes four parallel branches with different layers. The modification of the Inception model produced higher efficiency. By fine-tuning its structure, we minimized unnecessary complexity and improved feature extraction. This modification resulted in faster training and reduced computing costs. Overall, the modified model demonstrates that architectural customization can enhance performance while maintaining the core characteristics of the original Inception architecture. The details of the model layers are provided in Table 3.

**Table 3:** Modified Inception-4-block model details.

	Layer	Name	Type	Stride	Details
Initial layers	1	Input	Image input	–	227 × 227 × 3
	2	Conv	2-D Convolution	One	32, 3 × 3 Convolution
	3	ReLU	ReLU	–	ReLU
Block 1	4	Conv	2-D Convolution	One	64, 1 × 1 Convolution
	5	Conv	2-D Convolution	One	32, 3 × 3 Convolution
	6	Conv	2-D Convolution	One	64, 1 × 1 Convolution
	7	MaxPool	2-D Max Pooling	One	3 × 3 Max Pooling
	8	BatchNorm	Batch Normalization	–	-
	9	ReLU	ReLU	–	ReLU
	10	ReLU	ReLU	–	ReLU
	11	BatchNorm	Batch Normalization	–	Batch Normalization
	12	Conv	2-D Convolution	One	32, 5 × 5 Convolution
	13	ReLU_	ReLU	–	ReLU
	14	Conv	2-D Convolution	One	32, 3 × 3 Convolution
	15	BatchNorm	Batch Normalization	–	Batch Normalization
	16	MaxPool	2-D Max Pooling	One	1 × 1 Max Pooling
	17	BatchNorm	Batch Normalization	–	Batch Normalization
	18	Addition	Addition	–	Element-wise Addition of 4 input
	19	Conv	2-D Convolution	Two	64, 2 × 2 Convolution
	20	ReLU	ReLU	–	ReLU
Block 2	21	Conv	2-D Convolution	One	128, 1 × 1 Convolution
	22	Conv	2-D Convolution	One	64, 1 × 1 Convolution
	23	Conv	2-D Convolution	One	128, 1 × 1 Convolution
	24	MaxPool	2-D Max Pooling	One	3 × 3 Max Pooling
	25	ReLU	ReLU	–	ReLU
	26	ReLU	ReLU	–	ReLU
	27	BatchNorm	Batch Normalization	–	Batch Normalization
	28	Conv	2-D Convolution	One	64, 3 × 3 Convolution
	29	BatchNorm	Batch Normalization	–	Batch Normalization
	30	MaxPool_	2-D Max Pooling	One	1 × 1 Max Pooling
	31	BatchNorm	Batch Normalization	–	Batch Normalization
	32	ReLU	ReLU	–	ReLU
	33	Conv	2-D Convolution	One	64, 5 × 5 Convolution
	34	BatchNorm	Batch Normalization	–	Batch Normalization
	35	Addition	Addition	–	Element-wise Addition of 4 input
	36	Conv	2-D Convolution	One	128, 3 × 3 Convolution
	37	ReLU	ReLU	–	ReLU
Block 2	38	Conv	2-D Convolution	One	256, 1 × 1 Convolution
	39	Conv	2-D Convolution	One	128, 1 × 1 Convolution
	40	Conv	2-D Convolution	One	256, 1 × 1 Convolution
	41	MaxPool	2-D Max Pooling	One	3 × 3 Max Pooling
	42	BatchNorm	Batch Normalization	–	Batch Normalization
	43	ReLU	ReLU	–	ReLU
	44	BatchNorm	Batch Normalization	–	Batch Normalization
	45	Conv	2-D Convolution	One	128, 3 × 3 Convolution
	46	ReLU	ReLU	–	ReLU
Block 3	47	MaxPool	2-D Max Pooling	One	1 × 1 Max Pooling
	48	BatchNorm	Batch Normalization	–	Batch Normalization
	49	BatchNorm	Batch Normalization	–	Batch Normalization
	50	Conv	2-D Convolution	One	128, 5 × 5 Convolution
	51	ReLU	ReLU	–	ReLU
	52	Addition	Addition	–	Element-wise Addition of 4 input
	53	Conv	2-D Convolution	Two	256, 2 × 2 Convolution
	54	ReLU	ReLU	–	ReLU
Block 3	55	Conv	2-D Convolution	One	512, 1 × 1 Convolution
	56	Conv	2-D Convolution	One	256, 1 × 1 Convolution
	57	Conv	2-D Convolution	One	512, 1 × 1 Convolution
	58	MaxPool	2-D Max Pooling	One	3 × 3 Max Pooling
	59	BatchNorm	Batch Normalization	–	Batch Normalization
	60	BatchNorm	Batch Normalization	–	Batch Normalization
	61	Conv	2-D Convolution	One	256, 3 × 3 Convolution
	62	ReLU	ReLU	–	ReLU
	63	Conv	2-D Convolution	One	256, 5 × 5 Convolution
	64	BatchNorm	Batch Normalization	–	Batch Normalization
	65	MaxPool	2-D Max Pooling	One	1 × 1 Max Pooling
	66	ReLU	ReLU	–	ReLU
	67	ReLU	ReLU	–	ReLU
	68	BatchNorm	Batch Normalization	–	Batch Normalization
	69	Addition	Addition	–	Element-wise Addition of 4 input
	70	Conv	2-D Convolution	Two	512, 3 × 3 Convolution
	71	ReLU	ReLU	–	ReLU
	72	Gapool	2–D Global Average Pooling Layer	–	2–D Global Average Pooling
	73	Fc	Fully Connected	–	Fully Connected Layer
	74	Softmax	Softmax	–	Softmax

#### Initial layers

The model starts with an input layer that accepts RGB images of size $\:227\:\times\:\:227\:\times\:\:3.$ Next, a 2-D convolutional layer with * 32* filters of size 3 × 3 and a stride of 1 extracts initial features. A ReLU activation is then used to introduce nonlinearity before the output is passed to the first Inception block.

#### Block 1

Block 1 contains four parallel branches. One branch starts with a $\:1\:\times\:\:1$ convolutional layer with 64 filters followed by a convolutional layer with 32 filters of size $\:3\:\times\:\:3$. Another branch performs a$\:\:1\:\times\:\:1$ convolution with 64 filters, while, in parallel, a 3 × 3 max pooling layer is applied with a stride of 1. These branches are batch normalized. Some of the paths employ ReLU activation, while others employ batch normalization prior to moving forward with a $\:5\:\times\:\:5$ convolutional layer with 32 filters. Another path has a $\:3\:\times\:\:3$ convolutional layer with 32 filters. The last path has a $\:1\:\times\:\:1$ max pooling layer followed by batch normalization. The output of all four paths is subsequently combined via an element-wise addition layer. The output feature map is fed into a $\:2\:\times\:\:2$ convolutional layer of 64 filters and stride 2, followed by a ReLU activation to complete Block 1.

#### Block 2

In continuation of Block 1, a 2-D convolutional layer with 128 filters of size $\:1\:\times\:\:1$ and a stride of 1 is applied. In the next step, a $\:3\:\times\:\:3\:$ max pooling layer with a stride of 1 is used. This is followed by two ReLU activation layers to introduce nonlinearity and a batch normalization layer with offset and scale parameters of size $\:1\:\times\:\:1\:\times\:\:64$ to stabilize training. Subsequently, another 2-D convolutional layer with 64 filters of size 3 × 3 and a stride of 1 is applied, using weights of size $\:1\:\times\:\:1\:\times\:\:128$ and a bias of size $\:1\:\times\:\:1\:\times\:\:64.$ A second batch normalization layer with the same scale and offset dimensions is then employed. Following this, a$\:\:1\:\times\:\:1$ max pooling operation with a stride of 1 is applied, along with another batch normalization layer and a ReLU activation function. Finally, Block 2 includes a 2-D convolutional layer with 64 filters of size 5 × 5 and a stride of 1, using weights of size $\:5\:\times\:\:5\:\times\:\:128$ and biases of size $\:1\:\times\:\:1\:\times\:\:64$, followed by a batch normalization layer with offset and scale dimensions of $\:1\:\times\:\:1\:\times\:\:64$.

#### Block 3

Block 3 further increases the network depth and begins with three sequential $\:1\:\times\:\:1$ convolutional layers with $\:256,\:128$, and * 256* filters, respectively, followed by a $\:3\:\times\:\:3$ max pooling layer. These paths are processed using batch normalization and ReLU activation functions. A $\:3\:\times\:\:3\:$convolutional layer with * 128* filters is then applied, followed by a max pooling operation and additional normalization layers. To enhance spatial feature extraction, a $\:5\:\times\:\:5$ convolutional layer with * 128* filters is employed, followed by a ReLU activation function. The outputs of the branches in this block are combined using element-wise addition. Block 3 concludes with a $\:2\:\times\:2\:$down-sampling convolutional layer with * 256* filters and a stride of 2, followed by a ReLU activation.

#### Block 4

Block 4 is the final Inception block and begins with three sequential $\:1\:\times\:\:1$ convolutional layers with $\:512,\:256,$ and * 512* filters, respectively. A $\:3\:\times\:\:3$ max pooling layer operates in parallel with these convolutional paths. The resulting feature maps are batch normalized before being passed to a $\:3\:\times\:\:3$ convolutional layer with $\:256\:$filters, followed by a ReLU activation. The processing then continues through a $\:5\:\times\:\:5$ convolutional layer with * 256* filters. Next, a $\:1\:\times\:\:1$ max pooling operation is applied, and the outputs are again processed using batch normalization and ReLU activation layers. The outputs from all branches are subsequently combined using element-wise summation. Finally, a $\:3\:\times\:\:3$ convolutional layer with 512 filters and a stride of $\:2\:$is applied for dimensionality reduction, followed by a ReLU activation.

#### Final layers

**Fig. 4:**
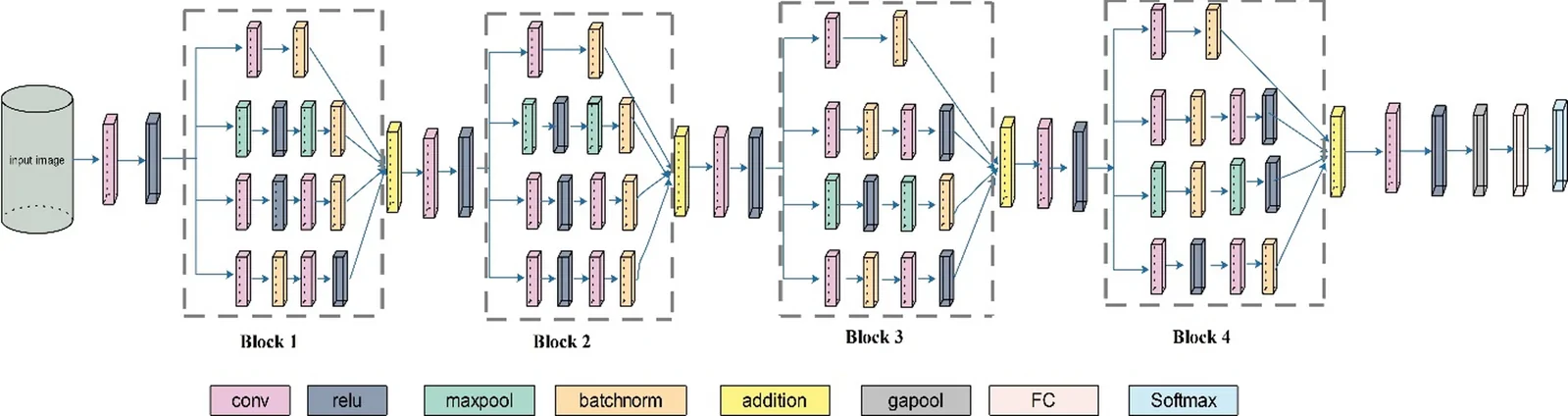
Architecture of the modified Inception-4-block model.

After the final Inception block, the model employs a global average pooling layer, where the spatial dimensions of each feature map are reduced to a single value. This is followed by a fully connected layer that maps the pooled * 512*-dimensional feature vector to the eight output classes. Finally, a softmax layer computes the class probabilities, completing the forward pass of the Inception model. The use of multiple hierarchical Inception blocks enables richer feature learning and more effective feature extraction compared to the standard Inception architecture. Figure 4 illustrates the architecture of the proposed Inception-4 block model.

### Customized ViT-3 model (three transformer encoder blocks)

The Vision Transformer (ViT) adapts the standard Transformer architecture — originally designed for sequential data — to handle 2D image inputs. As illustrated in Fig. 1, this is accomplished by decomposing an input image x ∈ R^(H×W×C) into a sequence of flattened 2D patches x_p ∈ R^(N×(P²·C)), where (H, W) denotes the spatial resolution of the original image, C is the number of channels, (P, P) is the resolution of each patch, and N = HW/P² is the total number of patches. This patch sequence constitutes the effective input length for the Transformer.

To project the patches into a consistent embedding space, each flattened patch is linearly mapped to a fixed-dimensional vector of size D using a trainable projection matrix, yielding the patch embeddings (Eq. 1). Following the design of BERT, a learnable classification token x_class is prepended to the patch embedding sequence. The state of this token at the final encoder layer serves as the global image representation y (Eq. 4), to which a classification head is attached during both pre-training and fine-tuning. Specifically, the classification head consists of a multi-layer perceptron (MLP) with one hidden layer during pre-training and is reduced to a single linear layer during fine-tuning.

To preserve spatial relationships among patches, standard learnable 1D positional embeddings are added to the patch embeddings. This straightforward positional encoding was found to be sufficient, as more complex 2D-aware alternatives did not yield notable performance improvements.

The resulting embedding sequence is then passed through the Transformer encoder, which is composed of alternating Multi-Head Self-Attention (MSA) and MLP blocks. Layer Normalization (LN) is applied prior to each block, and residual connections are incorporated after each block to facilitate stable gradient flow during training. The MLP within each encoder block comprises two fully connected layers with GELU activation. The forward pass of the encoder can be formally expressed as:

1 $${{\mathrm{Z}}_{\mathrm{0}}}={\mathrm{[}}{{\mathrm{X}}_{{\mathrm{Class}}}}{\mathrm{;X}}_{{\mathrm{p}}}^{{\mathrm{1}}}{\text{E; X}}_{{\mathrm{p}}}^{{\mathrm{2}}}{\mathrm{E;}} \ldots {\mathrm{X}}_{{\mathrm{p}}}^{{\mathrm{N}}}{\mathrm{E]}}+{{\mathrm{E}}_{{\mathrm{pos}}}}{\text{ E}} \in {{\mathrm{R}}^{{\mathrm{(}}{{\mathrm{P}}^{\mathrm{2}}}{\mathrm{.C)}} \times {\mathrm{D}}}},{\text{ }}{{\mathrm{E}}_{{\mathrm{pos}}}} \in {\text{ }}{{\mathrm{R}}^{{\mathrm{(N}}+{\mathrm{1)}} \times {\mathrm{D}}}}$$

2 $${Z^{\prime}}_{l}={\mathrm{MSA(LN(}}{{\mathrm{z}}_{l - 1}}{\mathrm{))}}+{{\mathrm{z}}_{l - 1,}}{\text{ l}}={\mathrm{1}} \ldots {\text{L }}$$

3 $$Zl={\mathrm{MLP(LN(}}z^{\prime}{_l}))+{{\mathrm{z}}^{{\prime}}}_{{l,}}{\text{ l}}={\mathrm{1}} \ldots {\mathrm{L}}$$

4 $${\mathrm{y}}={\mathrm{LN(}}{{\mathrm{Z}}^0}_{L}{\mathrm{)}}$$

In this study, we designed a customized ViT model that consists of three transformer encoder blocks. Each block included several layers. By customizing the ViT-3 transformer model instead of using the original ViT-12 transformer architecture, we achieved a more lightweight and computationally efficient network. We significantly reduced model depth and computational complexity. The ViT-3 transformer design decreased the number of parameters, resulting in faster training, lower memory consumption, and quicker inference. Despite the reduced architecture, the model retained the core self-attention mechanism necessary for capturing global contextual information.

The details of the transformer layers and activations along with channels, batch size and the number of patches are provided in Table 4.

**Table 4:** Details of ViT-3 model layers and activation functions.

	Layer	Name	Type	Activations
Transformer 1	1	Imageinput	Image input	384(S) × 384(S) × 3(C) ×1(B)
	2	Embedding	Patch embedding	576 (S) × 192(C) ×1(B)
	3	Re-flatten	Function	576 (S) × 192(C) ×1(B)
	4	Clsembed_concat	Embedding concatenation	577 (S) × 192(C) ×1(B)
	5	Posembed_input	Position embedding	577 (S) × 192(C) ×1(B)
	6	Add	Addition	577 (S) × 192(C) ×1(B)
	7	Dropout	Dropout	577 (S) × 192(C) ×1(B)
	8	Encoderblock1_layernorm	Layer normalization	577 (S) × 192(C) ×1(B)
	9	Encoderblock1_mha	Self-attention	577 (S) × 192(C) ×1(B)
	10	Encoderblock1_dropout	Dropout	577 (S) × 192(C) ×1(B)
	11	Encoderblock1_add	Addition	577 (S) × 192(C) ×1(B)
	12	Encoderblock1_layernorm	Layer normalization	577 (S) × 192(C) ×1(B)
	13	Encoderblock1_ conv1d	1-D convolution	577 (S) × 768(C) ×1(B)
	14	Encoderblock1_gelu	GELU	577 (S) × 768(C) ×1(B)
	15	Encoderblock1_dropout	Dropout	577 (S) × 768(C) ×1(B)
	16	Encoderblock1_conv1d	1-D convolution	577 (S) × 192(C) ×1(B)
	17	Encoderblock1_dropout	Dropout	577 (S) × 192(C) ×1(B)
	18	Encoderblock1_add	Addition	577 (S) × 192(C) ×1(B)
Transformer 2	19	Encoderblock2_layernorm	Layer normalization	577 (S) × 192(C) ×1(B)
	20	Encoderblock2_mha	Self-attention	577 (S) × 192(C) ×1(B)
	21	Encoderblock2_dropout	Dropout	577 (S) × 192(C) ×1(B)
	22	Encoderblock2_add	Addition	577 (S) × 192(C) ×1(B)
	23	Encoderblock2_layernorm	Layer normalization	577 (S) × 192(C) ×1(B)
	24	Encoderblock2_conv1d	1-D convolution	577 (S) × 768(C) ×1(B)
	25	Encoderblock2_gelu	GELU	577 (S) × 768(C) ×1(B)
	26	Encoderblock2_dropout	Dropout	577 (S) × 768(C) ×1(B)
	27	Encoderblock2_conv1d	1-D convolution	577 (S) × 192(C) ×1(B)
	28	Encoderblock2_dropout	Dropout	577 (S) × 192(C) ×1(B)
	29	Encoderblock2_add	Addition	577 (S) × 192(C) ×1(B)
Transformer 3	30	Encoderblock3_layernorm	Layer normalization	577 (S) × 192(C) ×1(B)
	31	Encoderblock3_mha	Self-attention	577 (S) × 192(C) ×1(B)
	32	Encoderblock3_dropout	Dropout	577 (S) × 192(C) ×1(B)
	33	Encoderblock3_add	Addition	577 (S) × 192(C) ×1(B)
	34	Encoderblock3_layernorm	Layer normalization	577 (S) × 192(C) ×1(B)
	35	Encoderblock3_conv1d	1-D convolution	577 (S) × 768(C) ×1(B)
	36	Encoderblock3_gelu	GELU	577 (S) × 768(C) ×1(B)
	37	Encoderblock3_dropout	Dropout	577 (S) × 768(C) ×1(B)
	38	Encoderblock3_conv1d	1-D convolution	577 (S) × 192(C) ×1(B)
	39	Encoderblock3_dropout	Dropout	577 (S) × 192(C) ×1(B)
	40	Encoderblock3_add	Addition	577 (S) × 192(C) ×1(B)
	41	Encoder_norm	Layer normalization	577 (S) × 192(C) ×1(B)
	42	Cls_index	1-D Indexing	192(C) ×1(B)
	43	Head	Fully connected	8(C) ×1(B)
	44	Softmax	Softmax	8(C) ×1(B)

#### Initial embedding and preprocessing

The model begins with an Image Input layer that receives images of size $\:384\:\times\:\:384\:\times\:\:3$. The input is passed to a Patch Embedding layer, which divides the image into 576 patches and embeds each patch into a 192-dimensional vector, producing an output of $\:576\:\times\:\:192\:\times\:\:1$. This representation is maintained by a re-flatten function. Next, [CLS] token concatenation prepends a class token, increasing the sequence length to $\:577\:\times\:\:192\:\times\:\:1.$ A position embedding is then incorporated to preserve positional information. The resulting representation is passed through an addition layer to combine token and positional embeddings, followed by a dropout layer for regularization.

#### Transformer 1

The first transformer encoder begins with a Layer Normalization to normalize the input sequence. It follows that with a Multi-Head Self-Attention (MHSA) mechanism such that the model can attend to various spatial locations for all the patches. A dropout layer is utilized after the attention stage, followed by a residual connection that combines the attention output with the initial input. A second Layer Normalization is then applied, after which the feature dimension is expanded from $\:192\:\boldsymbol{t}\boldsymbol{o}\:768$ through a 1D convolution. This is followed by a GELU activation function and dropout for regularization. A second 1D convolution reduces the feature dimension back to * 192,* followed by a final dropout and residual addition, completing the first transformer encoder block.

#### Transformer 2

Similar to Transformer Block 1, Transformer Block 2 starts with Layer Normalization and MHSA, followed by dropout and residual addition. The block then applies another normalization layer and a 1D convolution that increases the feature dimensions to * 768*. This is followed by a GELU activation, dropout, and a second 1D convolution to bring it back to the original * 192* dimensions. The block ends with a final dropout and residual addition.

#### Transformer 3

Transformer Block 3 follows the same structure as the first two blocks. It begins with Layer Normalization and MHSA, followed by dropout and residual addition. Another normalization layer is applied, and the features are processed through 1D convolutions $\:(192\:\to\:\:768\:\to\:\:192),$ separated by GELU activation and dropout layers. The block ends with a residual addition, completing the third and final encoder block.

#### Classification Head

**Fig. 5:**
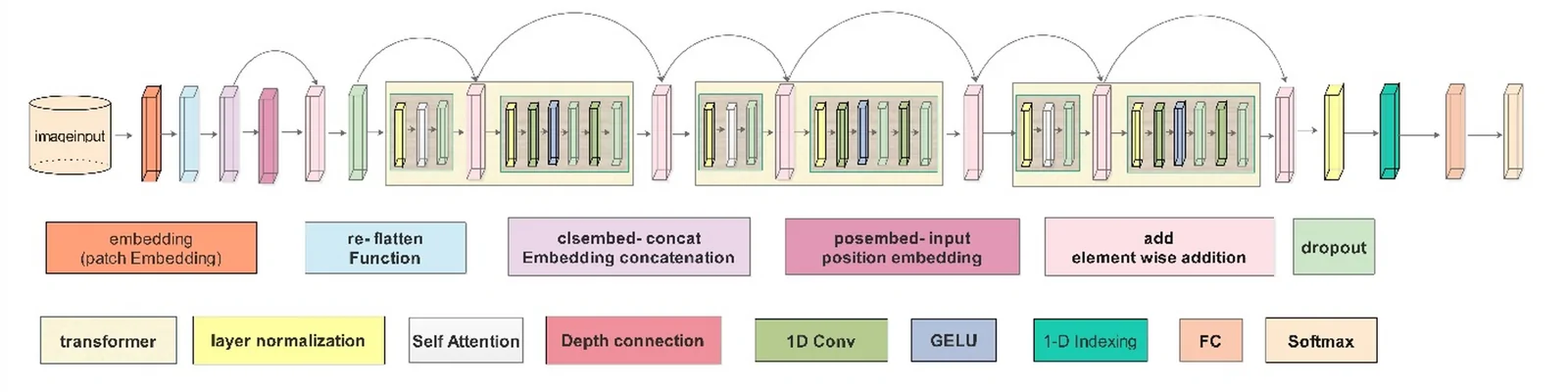
Architecture of customized ViT-3 model.

After processing the data by all three transformer blocks, a final Layer Normalization is conducted. The output **for** the [CLS] token is then taken via a 1D indexing operation, and the sequence is collapsed into a single 192-dimensional vector. This is then fed through a fully connected layer that projects it to $\:8\:$classes. The softmax activation function is then applied to produce class probabilities. The architecture of ViT 3 Transformer is presented in Fig. 5.

### Feature fusion and selection

Feature fusion refers to the process of integrating information from multiple sources into a single representation in order to enhance discriminative capability^53^. After extracting deep feature vectors from both the customized ViT and the modified Inception-4-block model, a weighted fusion technique was employed to combine the complementary features of both networks for GI image classification.

Let there be two feature vectors $\:{\mathbf{F}}_{\mathbf{V}\mathbf{i}\mathbf{T}\:}{\in\:\mathbb{R}}^{\boldsymbol{N}\:\times\:192}$ and $\:{\boldsymbol{F}}_{\boldsymbol{I}\boldsymbol{n}\boldsymbol{c}\boldsymbol{e}\boldsymbol{p}}$ $\:{\in\:\mathbb{R}}^{\boldsymbol{N}\:\times\:512}$ denote the feature matrices obtained from the ViT and Inception block, respectively. Due to the differing dimensionalities of the two feature vectors, the Inception features were first projected into a $\:192\:$ dimensional space using a linear transformation as $\:{\boldsymbol{F}}^{\prime}_{\boldsymbol{I}\boldsymbol{n}\boldsymbol{c}\boldsymbol{e}\boldsymbol{p}}={\boldsymbol{F}}_{\boldsymbol{I}\boldsymbol{n}\boldsymbol{c}\boldsymbol{e}\boldsymbol{p}}\:$ W, where $\:\boldsymbol{W}\:{\in\:\mathbb{R}}^{512\times\:192}\:$ is a learnable projection matrix and $\:{\boldsymbol{F}}^{\prime}_{\boldsymbol{I}\boldsymbol{n}\boldsymbol{c}\boldsymbol{e}\boldsymbol{p}}{\in\:\mathbb{R}}^{\boldsymbol{N}\:\times\:192}$. Following dimensional alignment, the fused feature representation was calculated using a weighted fusion technique, as expressed in Eq. 1:

5 $$\:\:{F}_{fused}=\:{w}_{1}\:\cdot \:{F}_{ViT\:}+\:{w}_{2}\:\cdot \:{F}^{\prime}_{Incep}$$

where *w*_*1*_ and *w*_*2*_ are the fusion weights assigned to the ViT and Inception features, respectively, these weights satisfy the constraint given in Eq. 2:

6 $$\:{w}_{1}+\:{w}_{2}\:=\:1\:and\:{w}_{1},\:\:{w}_{2}\:\in\:\:[0,\:1].$$

The fusion weights were empirically adjusted to balance the contribution of global contextual features extracted by the ViT and local features extracted by the modified Inception-4-block model based on validation performance. We concatenated the *w*_*1*_ and *w*_*2*_ resulting total 704 features then selected the best 192 features. The resulting fused feature vector $\:{\mathbf{F}}_{\mathbf{f}\mathbf{u}\mathbf{s}\mathbf{e}\mathbf{d}}\:{\in\:\mathbb{R}}^{\boldsymbol{N}\:\times\:192}$ was then forwarded to the final classification stage. This weighted fusion method improved classification performance on GI image datasets by efficiently utilizing the balancing capabilities of both models.

### Tree growth algorithm (TGA)

The fused feature vector in this work may contain redundant information. Consequently, the Tree Growth Algorithm (TGA) is used to select the most significant features. The proposed algorithm is inspired by the competition among trees for sunlight and food^54^. The method categorizes the process into four groups: the best trees group, the competition for light group, the remove and replace group, and the reproduction group.

In the best trees group, trees grow further due to favorable development conditions. Since the amount of sunlight received is sufficient, their competition mainly focuses on food. In the competition for light group, trees move away from nearby trees under different angles to access more light. The remove and replace group represent weak trees that do not grow sufficiently; these trees are removed and replaced with new ones. In the reproduction group, the best trees start to reproduce and generate new plants as a result of favorable growth. Because reproduction occurs near the mother tree, the new plants inherit some characteristics of that area.

The Tree Growth Algorithm begins with initialization, where a population of candidate solutions is generated randomly within the search domain, the iteration counter is set to * t=1,* and the maximum iteration number Maxiter is defined. Each candidate solution represents a binary feature selection vector, where a value of 1 indicates that a feature is selected and 0 indicates it is excluded. The population size N is divided into four groups: N1 (best trees), N2 (competition for light), N3 (remove and replace), and N4 (reproduction), where N = N1 + N2 + N3 + N4.While $\:t<Maxiter,\:$the population is evaluated and sorted according to fitness values. The fitness function evaluates each candidate solution based on classification accuracy and the number of selected features, rewarding subsets that achieve high accuracy with fewer features. For all solutions in $\:{N}_{1}$, a local search is performed using Eq. 7:

7 $$\:{T}_{I}^{J+1}=\:\frac{{T}_{j}^{i}}{\theta\:}+r{T}_{j}^{i}\:$$

Equation 3 updates each solution in N1 by adjusting feature weights based on the tree’s age factor θ and a random nutrient absorption rate r ~ U (0,1). This simulates the growth of the best trees by refining their feature subsets locally around the current best solution. Here, *θ* is the rate at which trees lose power as a result of age, rapid growth, and a decrease in available food. Additional y, 𝑟 is $\:U\left(\mathrm{0,1}\right),$ where the tree’s roots are directed to absorb nutrients for growth, which is growing at a pace of $\:r{T}_{j}^{i}\:$ units, since the tree is satisfied with the light.

Move *N*_*2*_ responses to the distance between the best, closest answers at various α angles. This step simulates competition for light, where each tree in N2 repositions itself between two neighboring solutions to explore new regions of the feature space. The distance between selected trees and others is computed as in Eq. 8.

8 $$\:{d}_{1}=\:{\left(\sum\:_{i=1}^{N1+N2}{\left(\:{T}_{\:\:N2}^{J}-\:{T}_{I}^{J}\:\right)}^{2}\right)}^{\frac{1}{2}}\:\:\&\:{\:d}_{i}\:\left\{\begin{array}{c}{d}_{i}\:\:\:\:\:\:\:if{T}_{N2}^{j}\:\ne\:{T}_{i}^{j}\\\:\infty\:\:\:\:\:\:\:if{T}_{N2}^{j}if{\:\:\:=T}_{i}^{J}\end{array}\right.\:\:\:\:\:\:$$

Equation 9 calculates the Euclidean distance between candidate solutions in the feature space, identifying the two nearest neighbors x1 and x2 for each tree in N2. To obtain linear combination between 𝑥_*1*_ and 𝑥_*2*_, both are selected with the minimal 𝑑𝑖 as presented in Eq. 9.

9 $$\:y=\:\lambda\:{x}_{1}+(1-\:\lambda\:){x}_{2}$$

**Fig. 6:**
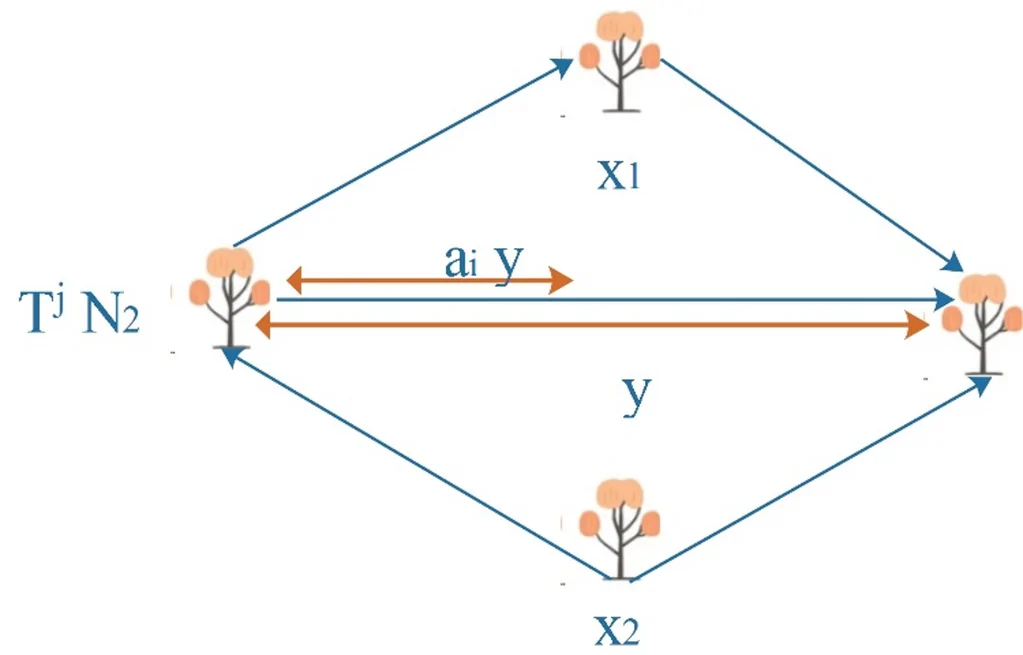
Moving between two adjacent trees.

Equation 10 computes a weighted linear interpolation between x1 and x2 using a random coefficient αi ~ U (0,1), allowing each N2 solution to move to a new position between its two closest neighbors. This mechanism encourages exploration of the search space while remaining guided by high-performing solutions. Lastly, as shown in Fig. 6, this tree may be moved between two nearby trees with 𝛼𝑖 = (0,1).

10 $$\:{T}_{{\:\:N}_{2}}^{j}=\:{T}_{{\:\:N}_{2}}^{j}+\:{a}_{i}y\:$$

**Fig. 7:**
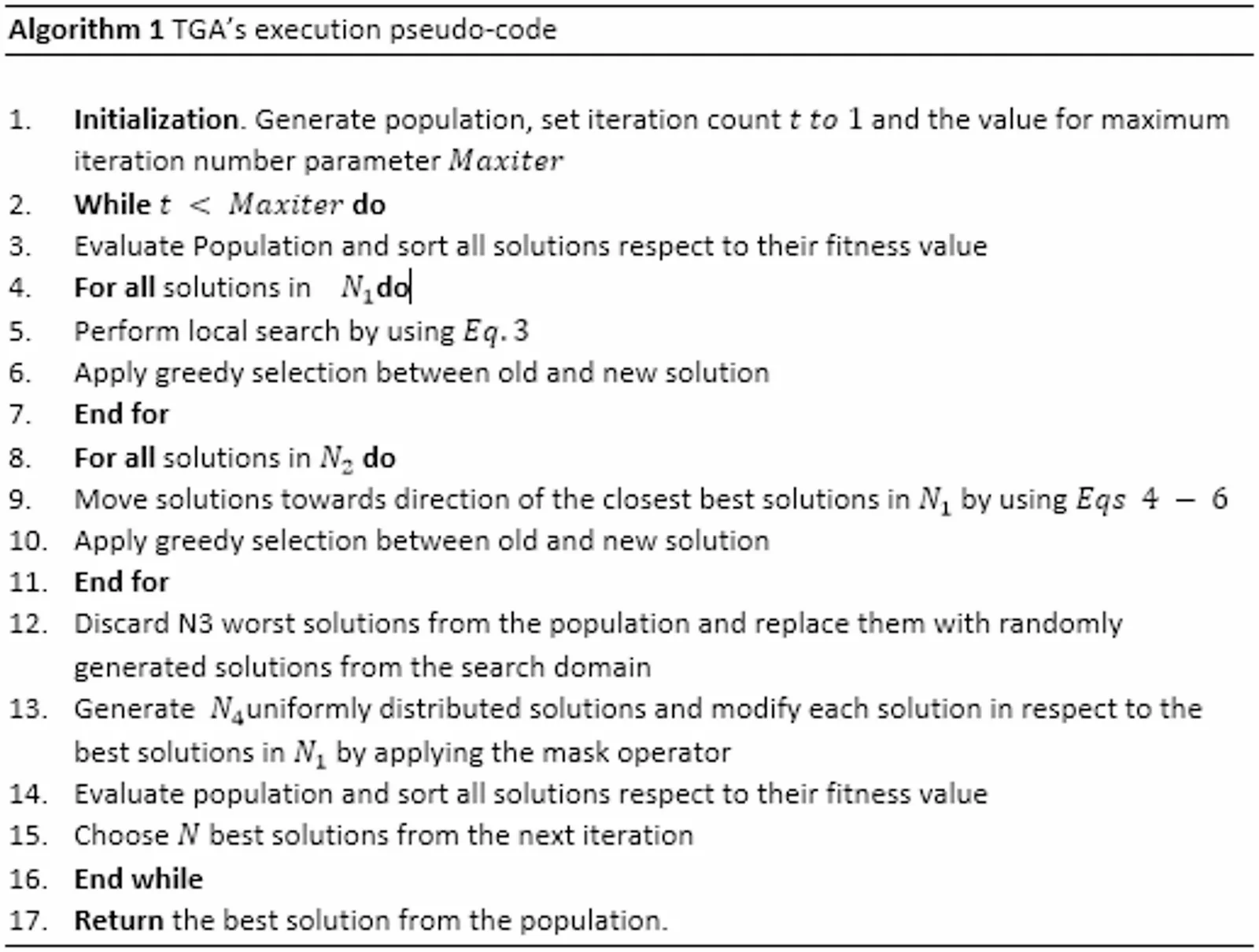
TGA’s execution pseudo-code.

Remove the *N*_*3*_ worst answer and adapt randomly generated ones. This step introduces diversity into the population by replacing the weakest candidate solutions with newly generated random feature subsets, preventing premature convergence. Generate a new population so that $\:N\:=\:{N}_{1}\:+\:{N}_{2}\:+\:{N}_{3}$.A new population (new population = new population $\:+\:N4$) is created, and each new answer is mixed with a best solution (of the population $\:{N}_{1}$) chosen at random. This reproduction step ensures that new candidate solutions inherit characteristics from high-performing parent solutions, accelerating convergence toward the optimal feature subset. Once the new population has been sorted, we take the number of initial population N from this new population as the starting population for the following iteration (based on the tournament, roulette wheel, or by selecting the best solutions). This iterative process continues until t = Maxiter, at which point the best candidate solution — the feature subset with the highest fitness value — is selected and passed to the downstream classifiers for final GI disease classification. The pseudo-code of the Tree Growth Algorithm used for feature selection is illustrated in Fig. 7.

## Results

In this section, the outcomes of the proposed framework are discussed. The remainder of this section includes the experimental setup, classification results, discussion, and comparison with previous work.

### Experimental setup

Various classifiers were used for classification, including Medium NN, Weighted NN, Quadratic SVM, Cubic SVM, MGSVM, Weighted KNN, and Fine KNN. The experiments were performed using MATLAB 2023b on a desktop PC.

### Evaluation metrics

The evaluation metrics, include accuracy, precision, sensitivity, and F1-score. To verify the proposed framework, the following experiments were conducted on each dataset. The datasets were split into training and testing sets in separate folders. For each class, 1,000 images were used for training while remaining images were used for testing.

### Training/testing setup/hyper parameters

The proposed system was evaluated on the Kvasir v1 and v2 datasets using various hyper parameters. Table 5 presents the hyper parameters and their values.

**Table 5:** Hyper parameters of ViT-3 & Inception Model.

Hyperparameters	Values	
	Inception model	ViT-3
Learning rate	0.0001	0.0005
Mini-batch size	20	32
Epochs	50	80
Optimizer	SGDM	SGDM
Weight learning rate factor	10	10
Bias learning rate factor	10	10

### Evaluation of the proposed work

For each dataset, the following experiments were conducted to verify the proposed framework:

1. Classification using the custom modified Inception-4-block model on both datasets (Kvasir v1 and Kvasir v2).
2. Classification using the customized ViT-3 model on both datasets (Kvasir v1 and Kvasir v2).
3. Classification using the proposed weighted fusion approach.
4. Feature selection for fused features using the TGA for classification.

The primary goal of these experiments is to evaluate how well the proposed framework performs, including:

a. Evaluating the performance of both models.

b. Determining the contribution of the fusion stage.

c. Assessing how the optimization process affects overall processing time.

These experiments were designed to address the above points, and the outcomes are presented in the following subsections.

### Evaluation using Kvasir v1 dataset

This section presents the classification results obtained on the Kvasir v1 dataset using different approaches. These include the customized ViT-3 model, the modified Inception-4-block model, a weighted fusion method that combines features from both models, and an optimization method designed to enhance performance. The results for all selected classifiers are reported in both numerical form and through confusion matrices. Seven classifiers were applied to the extracted features, namely Medium NN (MNN), Weighted NN (WNN), Quadratic SVM (QSVM), Cubic SVM (CSVM), MGSVM, Weighted KNN (WKNN), and Fine KNN (FKNN).

#### Results of modified Inception-4-block model

This section presents the classification performance of the modified Inception-4 block model on the Kvasir v1 dataset. The performance of seven classifiers was evaluated in terms of Accuracy, Sensitivity, Precision, F1-score, and Prediction Time and the results are presented in Table 6. Among the evaluated classifiers, the MNN classifier achieved an accuracy of 90.8% with a computation time of 61.125 s. It also attained a sensitivity of 90.78%, a precision of 90.8%, and an F1-score of 90.78%.

**Table 6:** Modified Inception-4-block model results.

Classifier	Sensitivity (%)	Precision (%)	F1 Score (%)	Accuracy (%)	Time(s)
MNN	90.78	90.8	90.78	90.8	61.125
WNN	93.57	93.57	93.57	93.6	76.024
QSVM	92.47	92.52	92.49	92.5	58.881
CSVM	96.45	96.47	96.45	96.5	57.958
MGSVM	91.56	91.68	91.61	91.6	68.269
WKNN	94.27	94.32	94.29	94.3	43.371
**FKNN**	**98.17**	**98.16**	**98.16**	**98.2**	**27.489**

**Fig. 8:**
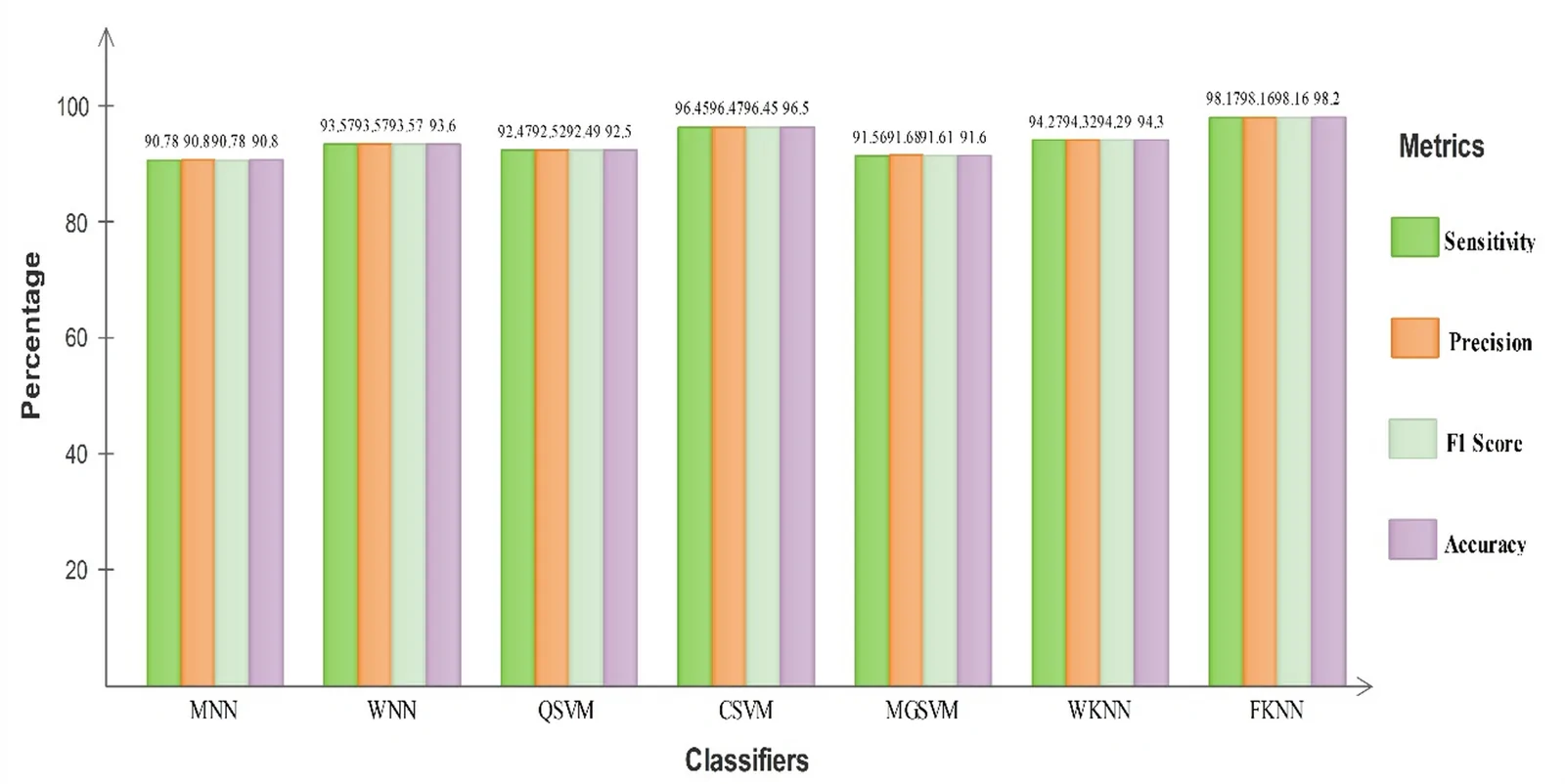
Graph comparing percentage results of accuracy, precision, F1-score, and sensitivity for the modified Inception-4-block model.

The WNN classifier achieved an accuracy of 93.6% with an execution time of 76.024 s. QSVM also produced good results, achieving an accuracy of 92.5% in 58.881 s. CSVM outperformed the other classifiers, considering both accuracy and execution time, achieving a sensitivity of 96.45%, an accuracy of 96.5%, a precision of 96.47%, and an F1-score of 96.45% with a comparatively short execution time of 57.958 s. The MGSVM classifier required 68.269 s to execute and achieved an accuracy of 91.6%, a sensitivity of 91.56%, a precision of 91.68%, and an F1-score of 91.61%. At a computation time of 43.371 s, the WKNN classifier demonstrated a sensitivity of 94.27%, a precision of 94.32%, an F1-score of 94.29%, and an accuracy of 94.3% (Fig. 8).

**Fig. 9:**
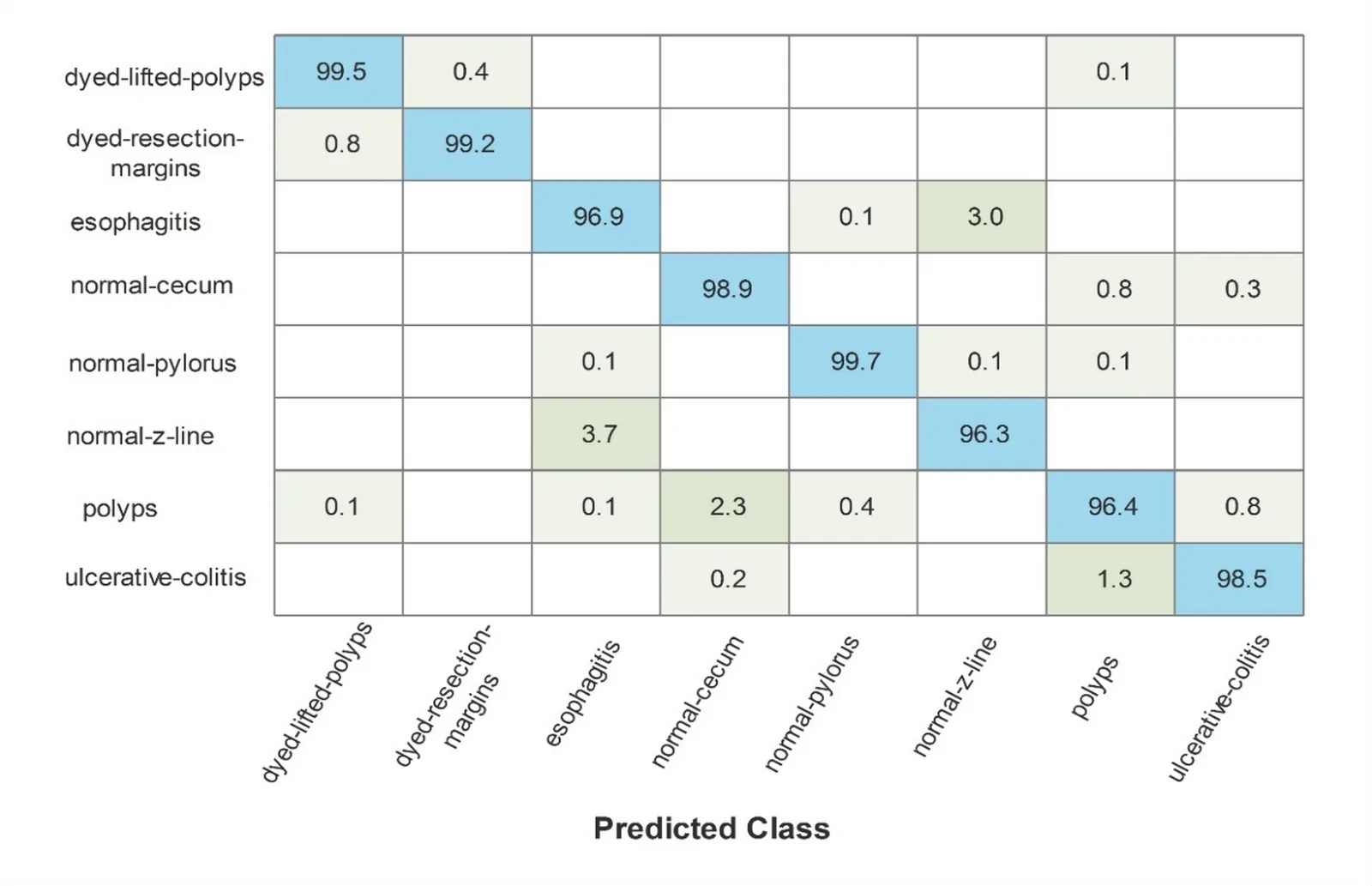
Confusion matrix of the proposed modified Inception-4-block model.

Furthermore, FKNN achieved the best overall performance with the fastest execution time of 27.489 s, along with a sensitivity of 98.17%, a precision of 98.16%, an F1-score of 98.16%, and an accuracy of 98.2%. The superior performance of FKNN is further illustrated by the confusion matrix shown in Fig. 9.

#### Results of Customized ViT-3 Model using Dataset Kvasir v1

**Fig. 10:**
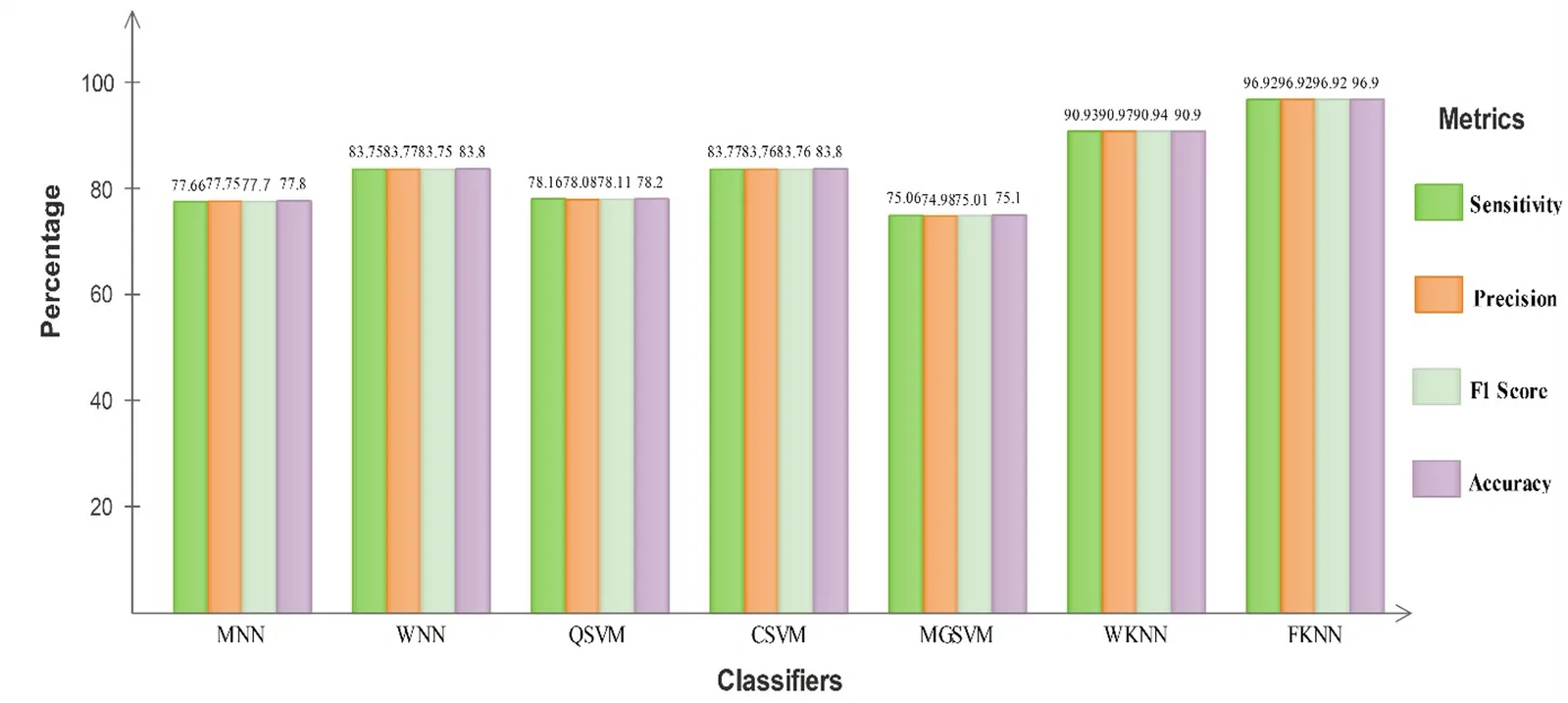
Graph comparing percentage results of accuracy, precision, F1-score, and sensitivity for the customized ViT-3 model.

The performance of the customized ViT-3 model was evaluated on the Kvasir v1 dataset using several machine learning classifiers. Table 7 presents the classification results The classifiers were evaluated based on key performance metrics, including computation time, accuracy, sensitivity, precision, and F1-score. Figure 10 shows the classification results obtained using features extracted from the customized ViT-3 model. Among the evaluated classifiers, FKNN achieved the highest accuracy of 96.9%.

**Table 7:** Classification performance of the customized ViT-3 model on the Kvasir v1 dataset.

Classifier	Sensitivity (%)	Precision (%)	F1-score (%)	Accuracy (%)	Time (s)
MNN	77.66	77.75	77.70	77.8	47.798
WNN	83.75	83.77	83.75	83.8	103.46
QSVM	78.16	78.08	78.11	78.2	27.876
CSVM	83.77	83.76	83.76	83.8	52.465
MGSVM	75.06	74.98	75.01	75.1	17.724
WKNN	90.93	90.97	90.94	90.9	6.7998
**FKNN**	**96.92**	**96.92**	**96.92**	**96.9**	**6.288**

**Fig. 11:**
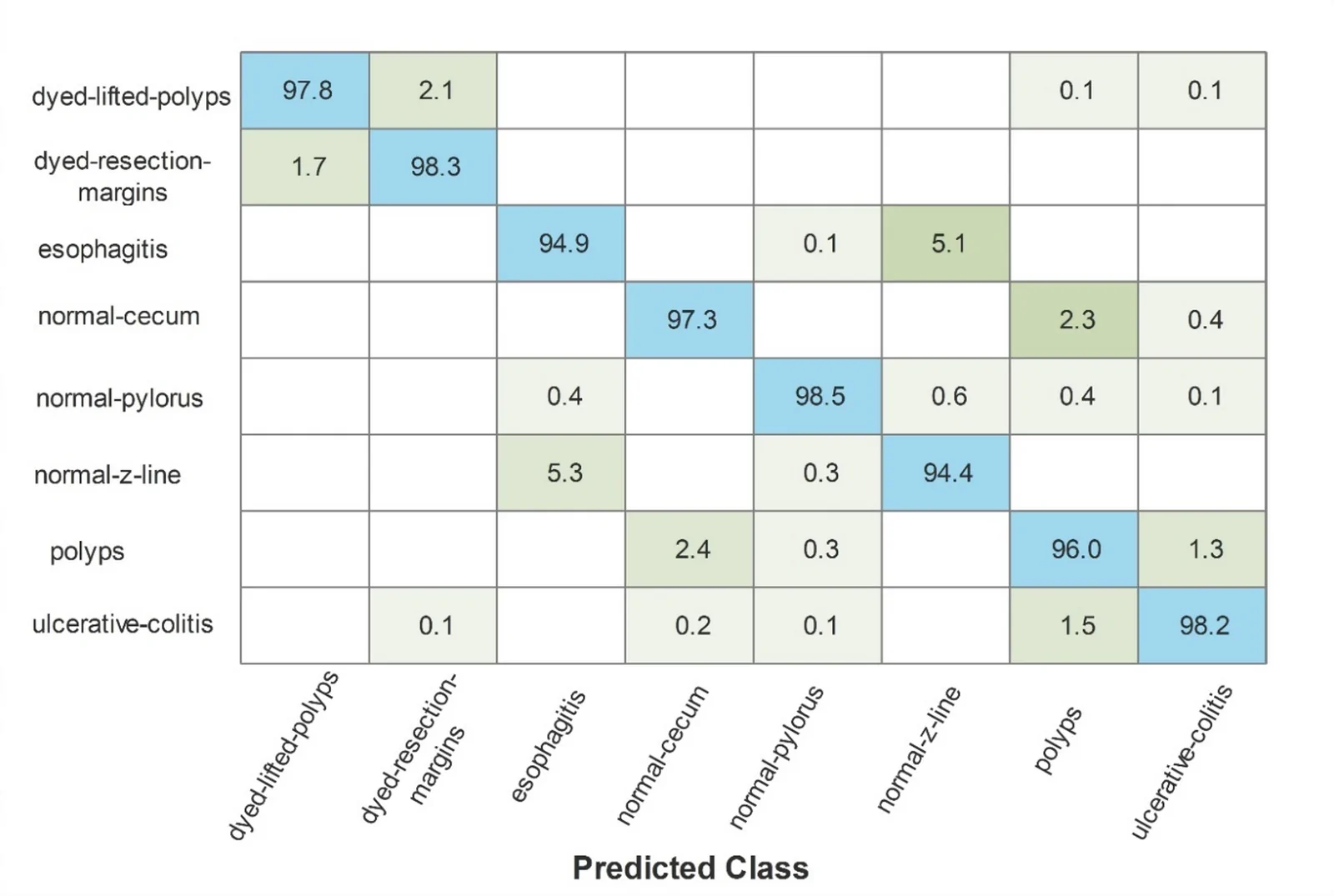
Confusion matrix of the proposed ViT-3 model.

FKNN outperformed all other classifiers, achieving the highest accuracy of 96.9% along with a sensitivity, precision, and F1-score of 96.92%. It also demonstrated the fastest computation time of 6.288 s, highlighting both its effectiveness and efficiency. The strong performance of FKNN is further validated by the confusion matrix shown in Fig. 11.

WKNN also showed competitive performance, achieving an accuracy of 90.9% with sensitivity and precision values of 90.93% and 90.97%, respectively, while maintaining a low computation time of 6.7998 s. This makes WKNN a suitable choice for applications where fast prediction is critical.

Both WNN and CSVM achieved comparable accuracy values of 83.8% with balanced F1-scores. However, WNN required significantly higher computation time (103.46 s) compared to CSVM (52.465 s). QSVM achieved an acceptable accuracy of 78.2% while demonstrating relatively fast execution at 27.876 s. Although MNN and MGSVM showed lower accuracy values of 77.8% and 75.1%, respectively, MGSVM achieved one of the shortest computation times at 17.724 s.

Overall, the results indicate that FKNN provides the best trade-off between classification accuracy and computational efficiency for the customized ViT-3 model on the Kvasir v1 dataset.

#### Results of fusion method using the Kvasir v1 dataset

**Fig. 12:**
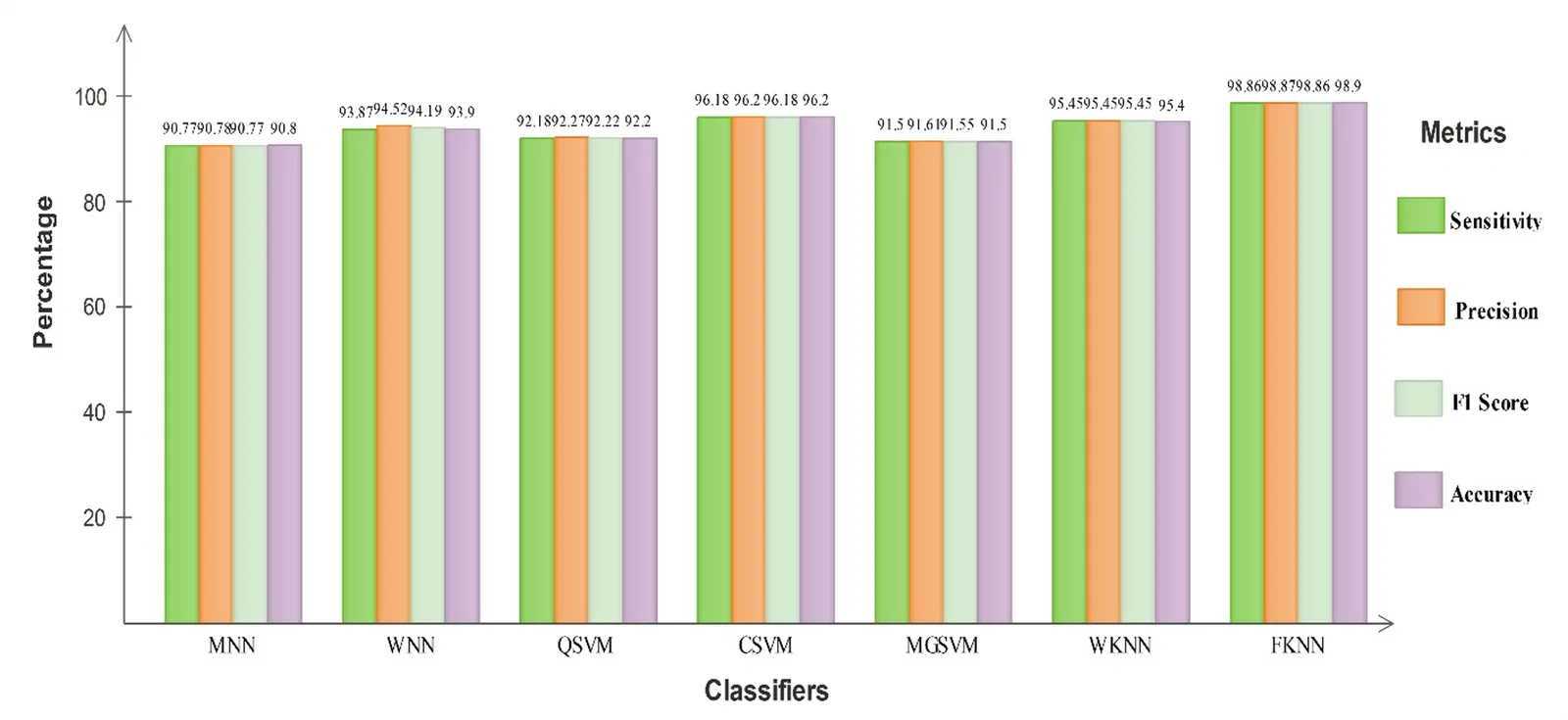
Visualization of percentage-based accuracy, precision, F1-score, and sensitivity results for the Weighted Fusion.

To improve classification accuracy, the weighted fusion method is used to combine the information obtained from the proposed models. The outcomes of this stage are presented in Table 8. The fusion results, obtained by combining features from the customized ViT-3 and the modified Inception-4-block model, provide a comparison across multiple classifiers. Among them, FKNN achieved the best overall performance with an accuracy of 98.9%, along with a sensitivity of 98.86%, a precision of 98.87%, and an F1-score of 98.86%. In addition, FKNN required only 7.42 s for prediction. The classifier performance after applying the weighted fusion method is shown in Fig. 12.

**Table 8:** Classification results of the weighted fusion model on the Kvasir v1 dataset.

Classifier	Sensitivity (%)	Precision (%)	F1-score (%)	Accuracy (%)	Time (s)
MNN	90.77	90.78	90.77	90.8	75.921
WNN	93.87	94.52	94.19	93.9	66.806
QSVM	92.18	92.27	92.22	92.2	23.112
CSVM	96.18	96.2	96.18	96.2	24.71
MGSVM	91.5	91.61	91.55	91.5	24.507
WKNN	95.45	95.45	95.45	95.4	7.0907
**FKNN**	**98.86**	**98.87**	**98.86**	**98.9**	**7.4152**

**Fig. 13:**
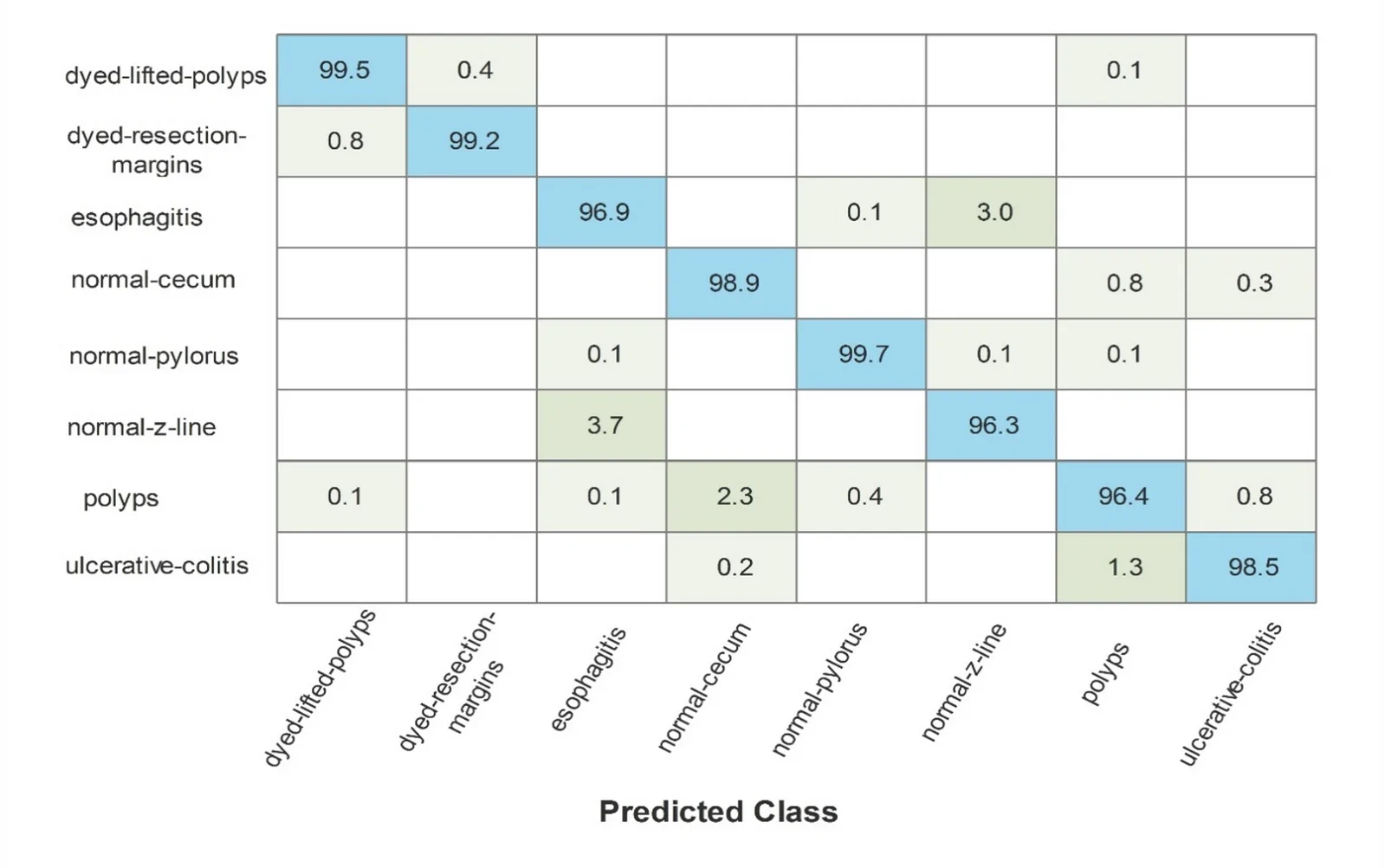
Confusion matrix of FKNN using the proposed fusion technique.

The confusion matrix in Fig. 13 provides further confirmation of FKNN’s performance. With a sensitivity, precision, and F1-score of 96.18% and an accuracy of 96.2%, the CSVM classifier placed in second.

WKNN also demonstrated strong performance, achieving an accuracy of 95.4% in 7.09 s, with sensitivity, precision, and F1-score values of 95.45%. WNN achieved an accuracy of 93.9% with an F1-score of 94.19%, but required a longer prediction time of 66.81 s. QSVM achieved an accuracy of 92.2% in 23.11 s with closely matched sensitivity and precision values. Competitive performance was also observed for MNN and MGSVM, which achieved accuracies of 90.8% and 91.5%, respectively; however, MNN required 75.92 s while MGSVM required 24.51 s. Overall, FKNN provided the best trade-off between accuracy and prediction time in the fusion stage.

#### Results of optimization method using the Kvasir v1 dataset

**Fig. 14:**
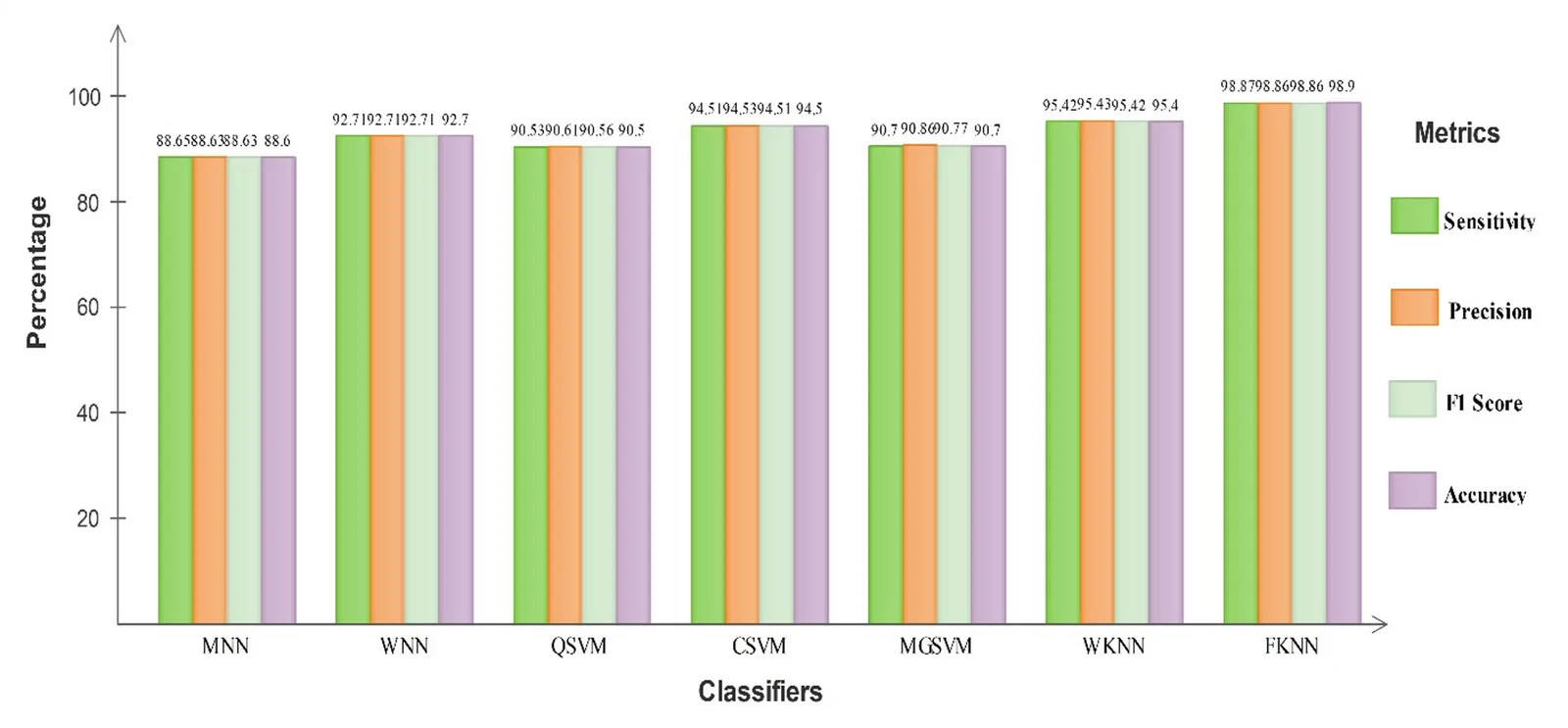
Graphical comparison of accuracy, precision, F1-score, and sensitivity percentages for the TGA optimization.

To evaluate the effect of feature optimization, TGA was applied to the fused feature set for classification on the Kvasir v1 dataset and the results are presented in Table 9. The performance of the classifiers is reported in terms of sensitivity, precision, F1-score, accuracy, and prediction time. Figure 14 represents the classification performance after applying the TGA optimization method.

**Table 9:** Performance after tree growth algorithm (TGA) optimization on the Kvasir v1 dataset.

Classifier	Sensitivity (%)	Precision (%)	F1-score (%)	Accuracy (%)	Time (s)
MNN	88.65	88.63	88.63	88.6	51.954
WNN	92.71	92.71	92.71	92.7	59.966
QSVM	90.53	90.61	90.56	90.5	17.996
CSVM	94.51	94.53	94.51	94.5	19.646
MGSVM	90.7	90.86	90.77	90.7	19.57
WKNN	95.42	95.43	95.42	95.4	3.1954
**FKNN**	**98.87**	**98.86**	**98.86**	**98.9**	**3.2058**

**Fig. 15:**
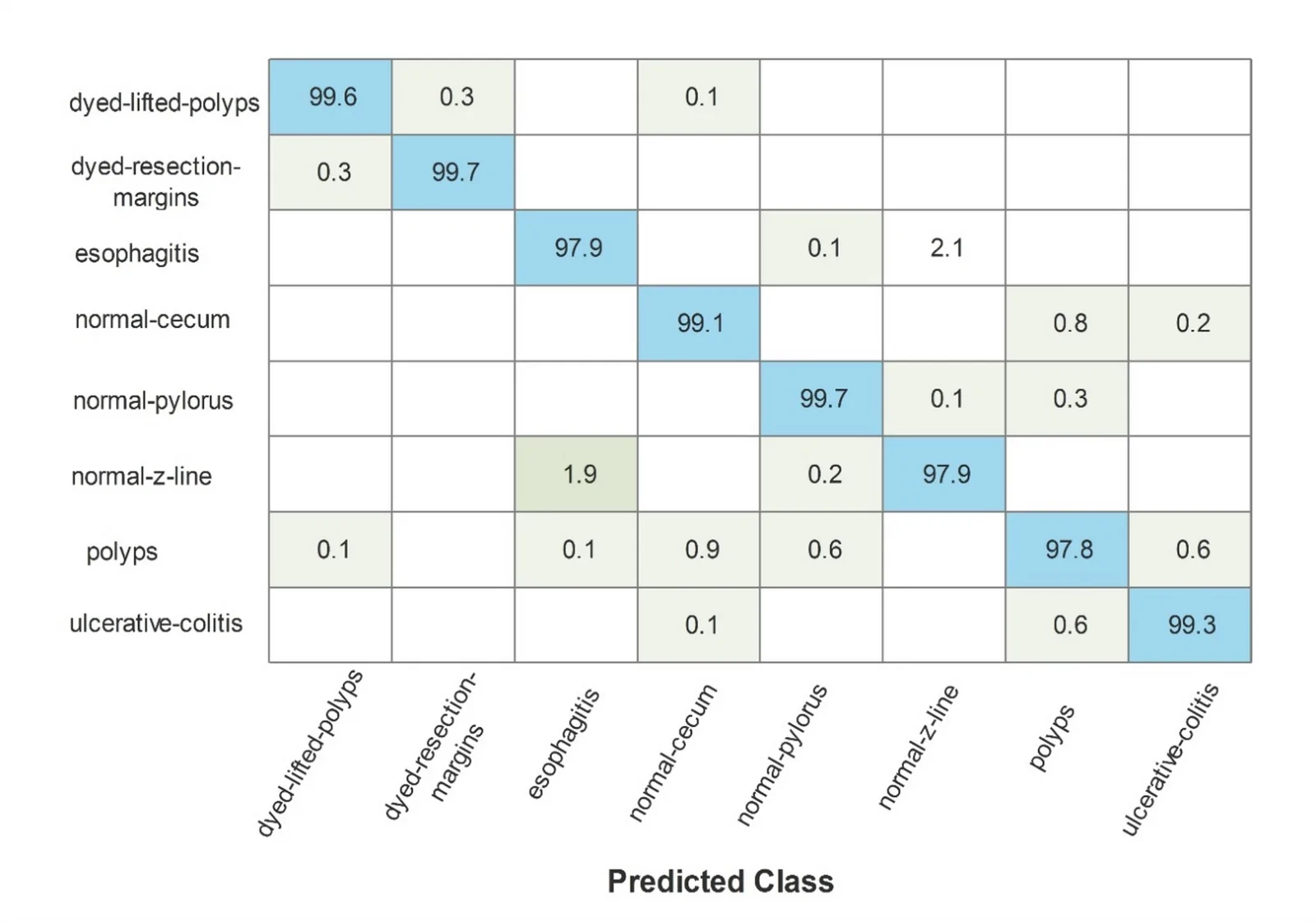
Confusion matrix of FKNN using the proposed optimization approach.

FKNN achieved the best overall performance after optimization, with an accuracy of 98.9% and the highest sensitivity (98.87%), precision (98.86%), and F1-score (98.86%). In addition, FKNN required only 3.21 s for prediction (Fig. 15).

WKNN also achieved strong performance, requiring approximately 3.20 s and attaining a sensitivity of 95.42%, a precision of 95.43%, and an accuracy of 95.4%. CSVM demonstrated competitive results with an accuracy of 94.5% in 19.65 s. QSVM achieved an accuracy of 90.5% in 17.99 s with sensitivity and precision values of 90.53% and 90.61%, respectively. MGSVM achieved an accuracy of 90.7% in 19.57 s. WNN achieved an accuracy of 92.7% with sensitivity, precision, and F1-score values of 92.71%, but required a longer prediction time of 59.97 s. MNN required 51.95 s and achieved an accuracy of 88.6%. Overall, FKNN and WKNN provided the most effective performance in terms of accuracy and prediction time after optimization.

**Fig. 16:**
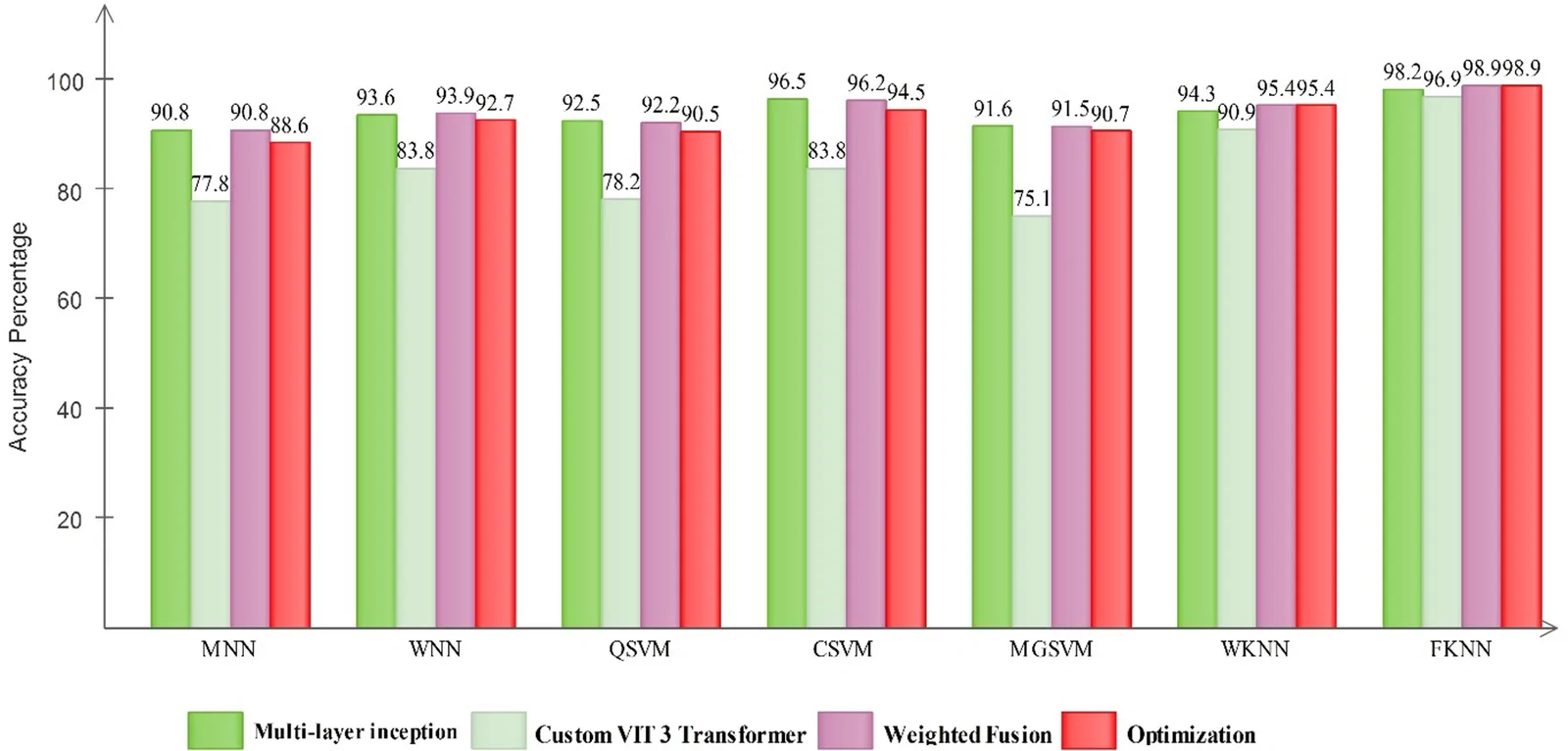
Prediction accuracy analysis based on classifiers for Kvasir v1 dataset.

**Fig. 17:**
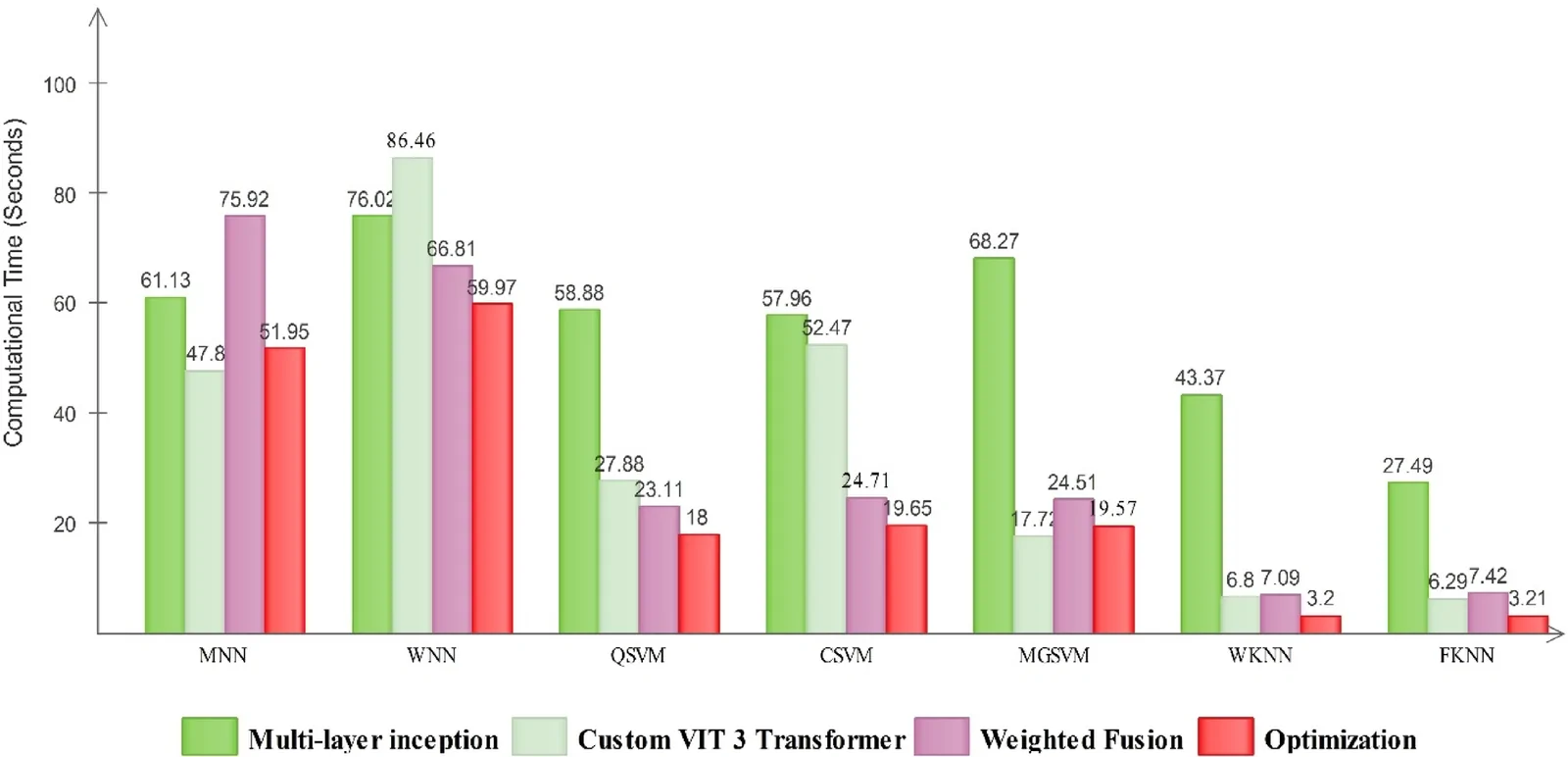
Prediction time analysis based on classifiers for Kvasir v1 dataset.

Figure 16 presents a comparison of prediction accuracy across all classifiers for the Kvasir v1 dataset. A brief comparison of the prediction time of each network on the Kvasir v1 dataset is presented in Fig. 17. From this figure, it can be observed that the WNN classifier exhibits the highest prediction time across all observations, reaching 86.46 s. In contrast, WKNN achieves the lowest prediction time of 3.2 s, making them suitable for real-time diagnostic support. The remaining classifiers show inference times ranging from 75.92 to 3.21 s.

### Evaluation using Kvasir v2 dataset

Similar experiments were performed for this dataset. These include the customized ViT-3 Transformer model and the modified Inception-4-block model, as well as a fusion method that combines the key features of both models and an optimization method designed to improve performance. In addition, confusion matrices are provided for the highest-performing classifiers to offer a detailed view of the classification results.

#### Results of modified Inception-4-block model using Kvasir v2 dataset

**Fig. 18:**
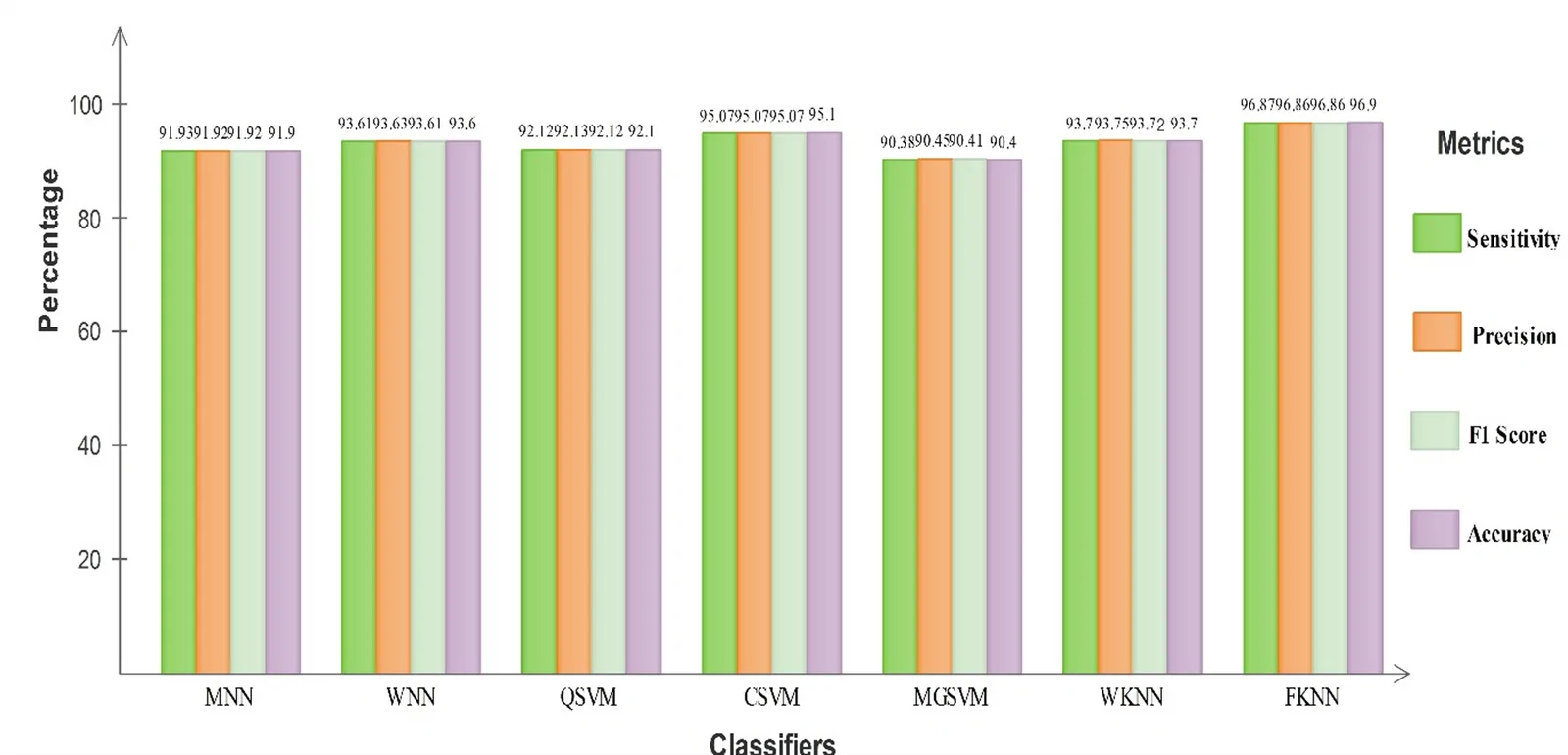
Graph comparing percentage results of accuracy, precision, F1-score, and sensitivity for the modified Inception-4-block model on the Kvasir v2 dataset.

Using the Kvasir v2 dataset, the performance of the modified Inception-4-block model was evaluated using several classifiers. Table 10 presents the results of this stage and the classifier performance is illustrated in Fig. 18. The model demonstrates consistent performance across datasets, with minor differences in accuracy. This slight variation is likely to be caused due to differences in dataset characteristics.

**Table 10:** Performance of the Modified Inception-4-block model on the Kvasir v2 dataset.

Classifier	Sensitivity (%)	Precision (%)	F1-score (%)	Accuracy (%)	Time (s)
MNN	91.93	91.92	91.92	91.9	78.114
WNN	93.61	93.63	93.61	93.6	68.58
QSVM	92.12	92.13	92.12	92.1	38.391
CSVM	95.07	95.07	95.07	95.1	38.366
MGSVM	90.38	90.45	90.41	90.4	42.508
WKNN	93.7	93.75	93.72	93.7	26.705
**FKNN**	**96.87**	**96.86**	**96.86**	**96.9**	**26.354**

**Fig. 19:**
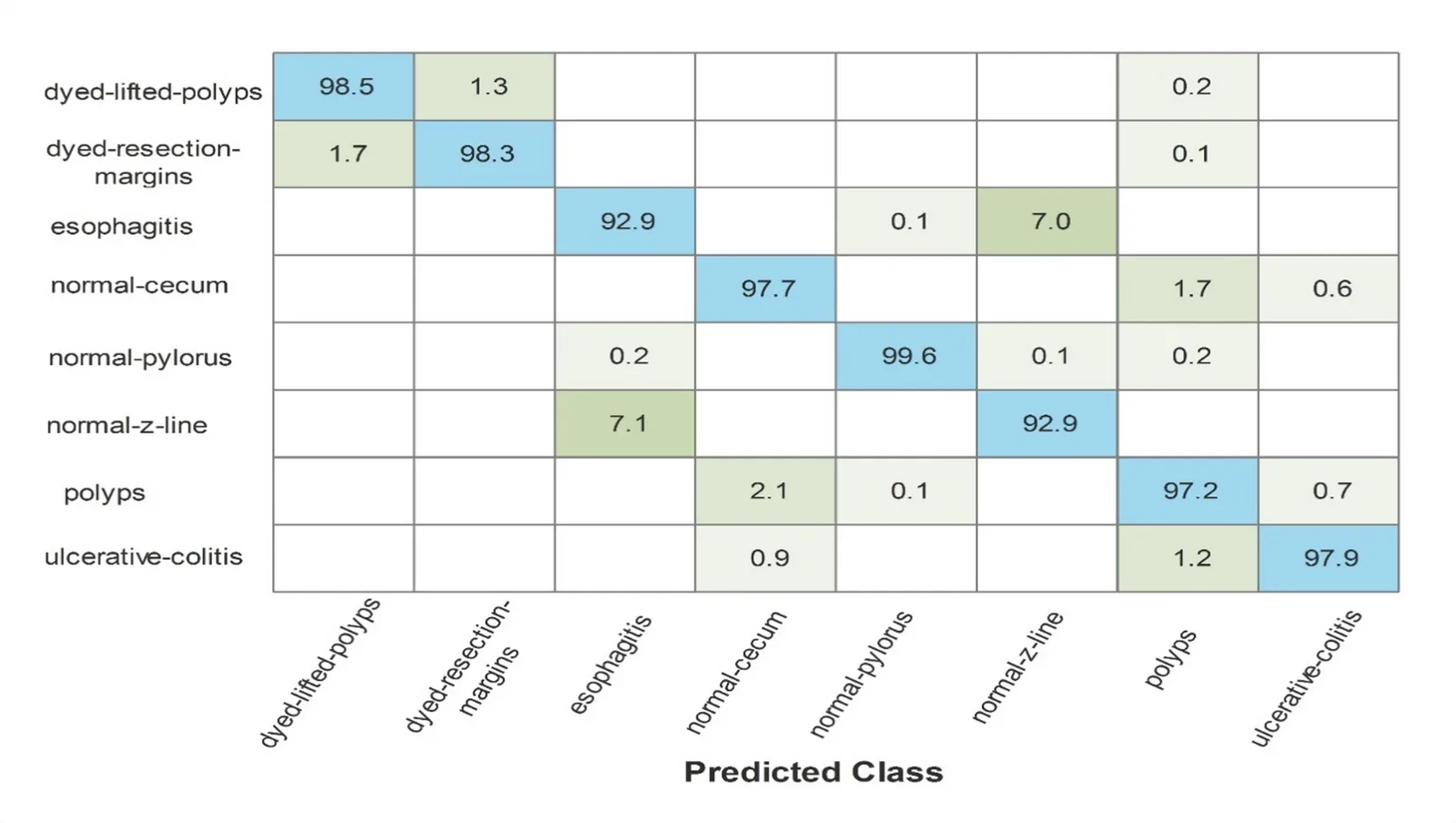
Confusion matrix of the proposed modified Inception-4-block model.

FKNN model performed best overall among the evaluated classifiers, achieving 96.87% sensitivity, 96.86% precision, 96.86% F1-score, and 96.9% accuracy, along with a comparatively fast computation time of 26.354 s. As shown in the confusion matrix in Fig. 19, most disease categories are correctly detected. This suggests that the learned feature representation captures meaningful structural patterns.

With a computation time of 38.366 s, the CSVM classifier ranked second, achieving sensitivity, precision, F1-score, and accuracy values of 95.07%. Other classifiers, such as WNN and WKNN, also demonstrated strong performance, with accuracy values of 93.6% and 93.7%, respectively, though with different processing efficiencies. MNN and QSVM achieved accuracies of 91.9% and 92.1%, respectively. MGSVM exhibited the lowest performance, with an accuracy of 90.4%. Overall, FKNN proved to be the most effective classifier for this dataset, providing high accuracy along with relatively fast computation time.

#### Results of customized ViT-3 transformer model using Kvasir v2 dataset

**Fig. 20:**
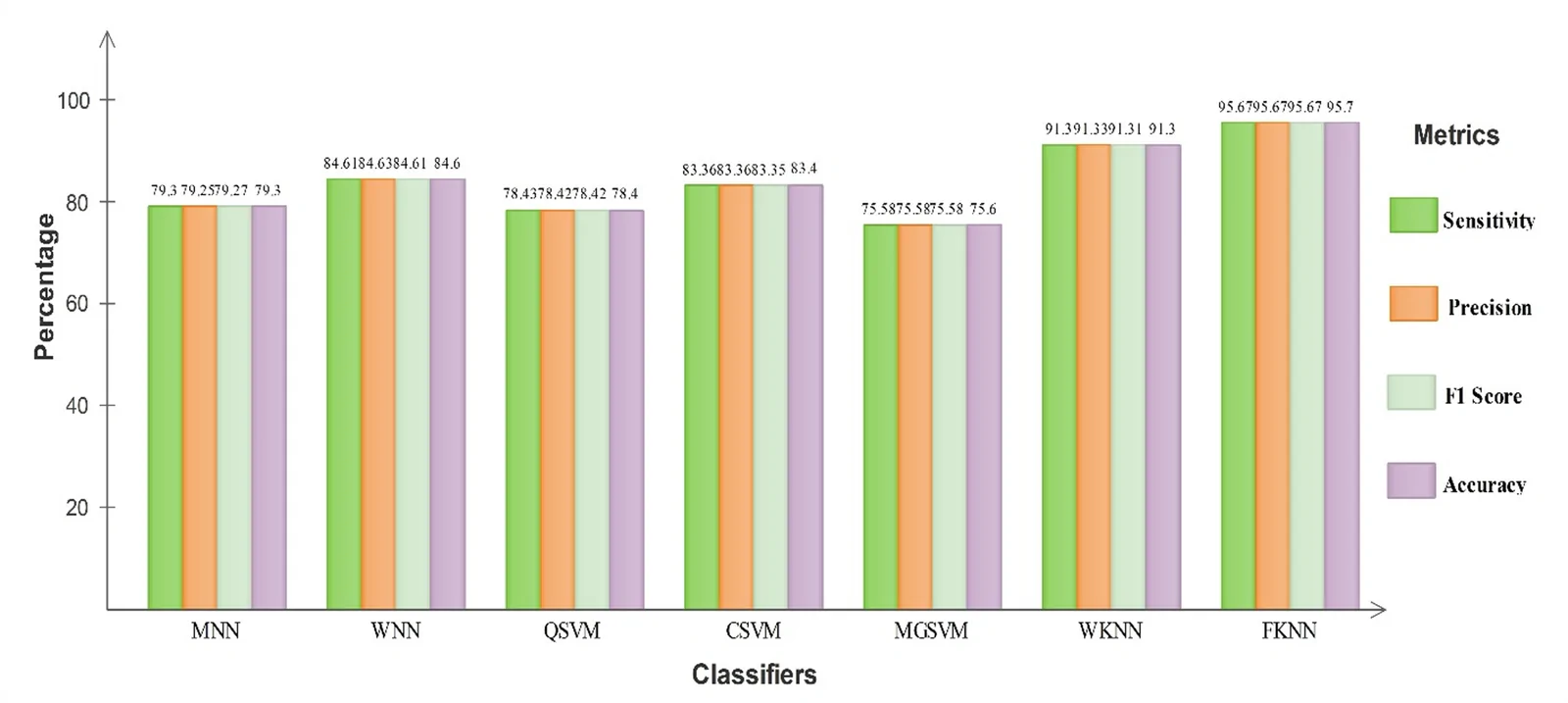
Graphical comparison of accuracy, precision, F1-score, and sensitivity percentages for the customized ViT-3 Transformer.

Using the Kvasir v2 dataset, the performance of the customized ViT-3 model was evaluated across multiple classifiers, as summarized in Table 11 and illustration of the results is provided in Fig. 20. Similar to the observations on Kvasir v1, the transformer-based model provides competitive results.

**Table 11:** Performance of the customized ViT-3 model on the Kvasir v2 dataset.

Classifier	Sensitivity (%)	Precision (%)	F1-score (%)	Accuracy (%)	Time (s)
MNN	79.3	79.25	79.27	79.3	40.724
WNN	84.61	84.63	84.61	84.6	81.914
QSVM	78.43	78.42	78.42	78.4	25.566
CSVM	83.36	83.35	83.35	83.4	49.909
MGSVM	75.58	75.58	75.58	75.6	16.706
WKNN	91.3	91.33	91.31	91.3	5.6041
**FKNN**	**95.67**	**95.67**	**95.67**	**95.7**	**5.7205**

**Fig. 21:**
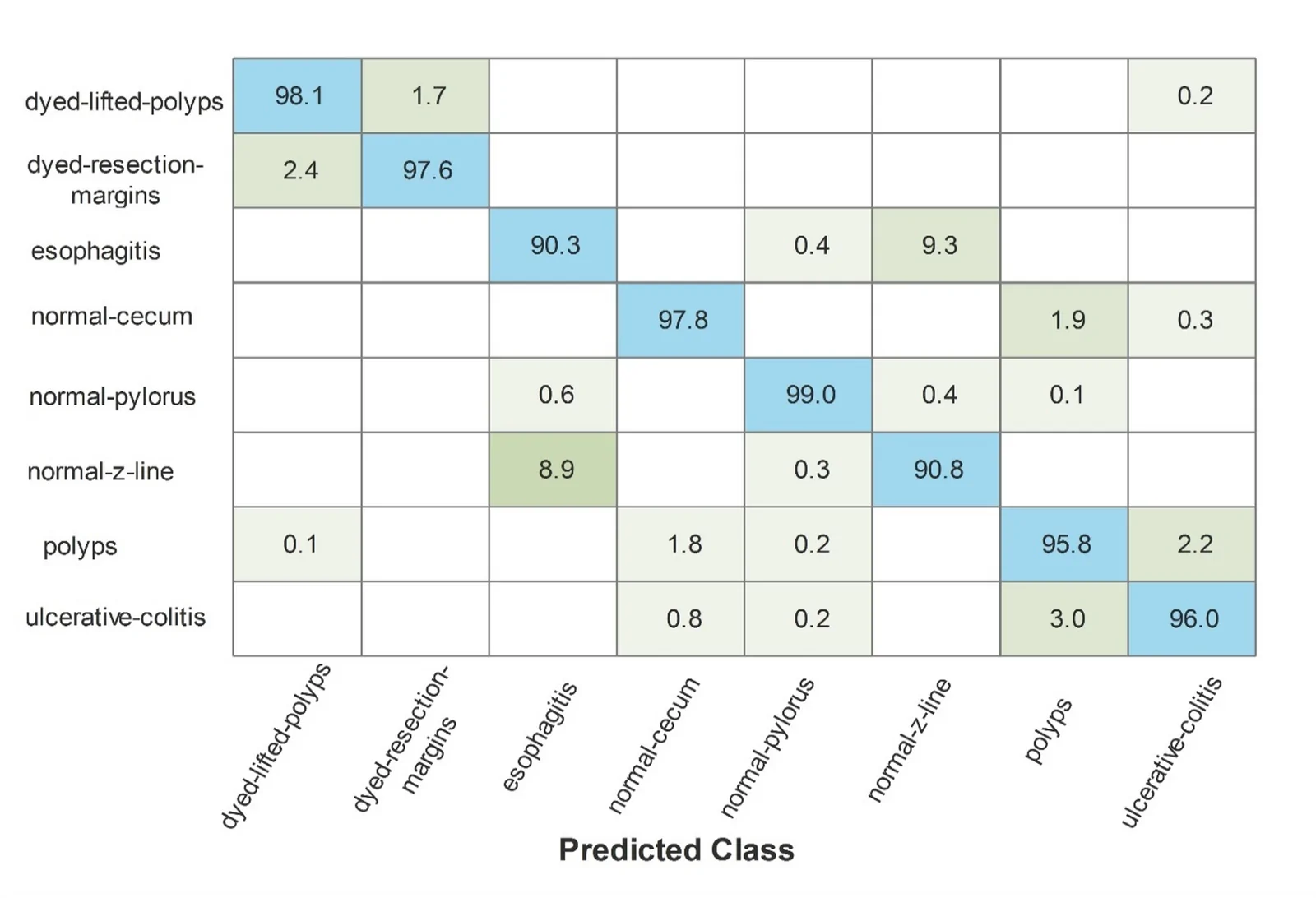
Confusion matrix of proposed ViT-3 model.

FKNN classifier achieved the highest performance, with 95.67% sensitivity, 95.67% precision, 95.67% F1-score, and 95.7% accuracy, along with a computation time of 5.7205 s. Figure 21 presents the corresponding confusion matrix. WKNN classifier ranked second, attaining 91.3% accuracy with a computation time of 5.6041 s. The WNN model achieved an accuracy of 84.6% but required substantially longer processing time (81.914 s). CSVM and QSVM demonstrated moderate performance, achieving accuracies of 83.4% and 78.4%, respectively, with higher computation times compared to the k-NN classifiers. Although MGSVM exhibited the shortest computation time (16.706 s), it recorded the lowest accuracy at 75.6%. The MNN classifier achieved an accuracy of 79.3%.

Overall, FKNN provided the best performance for the customized ViT-3 model on the Kvasir v2 dataset in terms of both accuracy and computational efficiency.

#### Results of fusion method using Kvasir v2 dataset

**Fig. 22:**
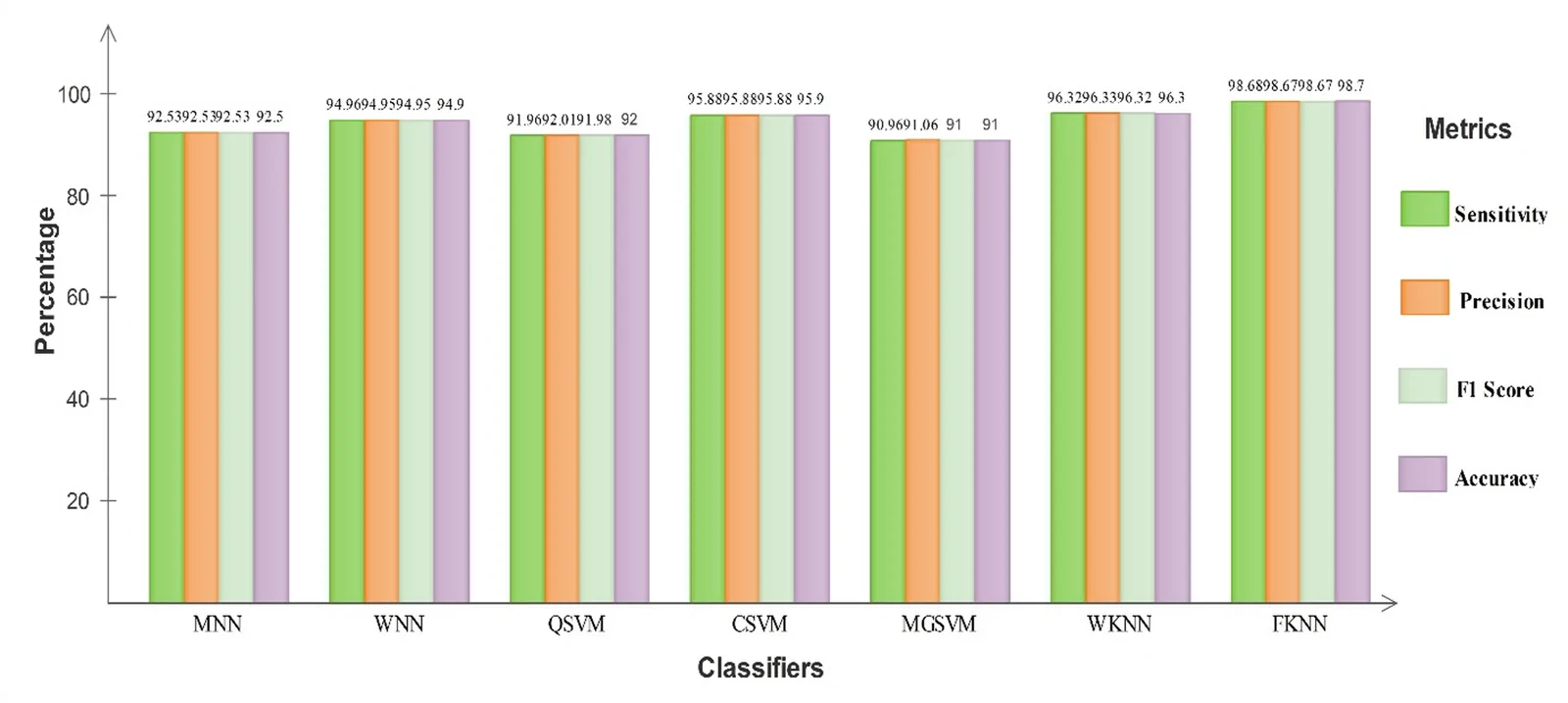
Visualization of percentage-based accuracy, precision, F1-score, and sensitivity results for the weighted fusion model on Kvasir v2 dataset.

To improve accuracy, the weighted fusion approach is utilized to combine information from both proposed models. The outcomes obtained by fusing features from the customized ViT-3 Transformer and the modified Inception-4-block model are presented in Table 12, providing a comparative evaluation across multiple classifiers. Figure 22 shows the classifier performance after applying the weighted fusion approach. The fusion approach again improves performance. This demonstrates that combining complementary feature representations can enhance robustness across different datasets.

**Table 12:** Performance of the weighted fusion model on the Kvasir v2 dataset.

Classifier	Sensitivity (%)	Precision (%)	F1-score (%)	Accuracy (%)	Time (s)
MNN	92.53	92.53	92.53	92.5	86.693
WNN	94.96	94.95	94.95	94.9	97.355
QSVM	91.96	92.01	91.98	92.0	26.894
CSVM	95.88	95.88	95.88	95.9	27.959
MGSVM	90.96	91.06	91.00	91.0	28.448
WKNN	96.32	96.33	96.32	96.3	8.9419
**FKNN**	**98.68**	**98.67**	**98.67**	**98.7**	**9.035**

**Fig. 23:**
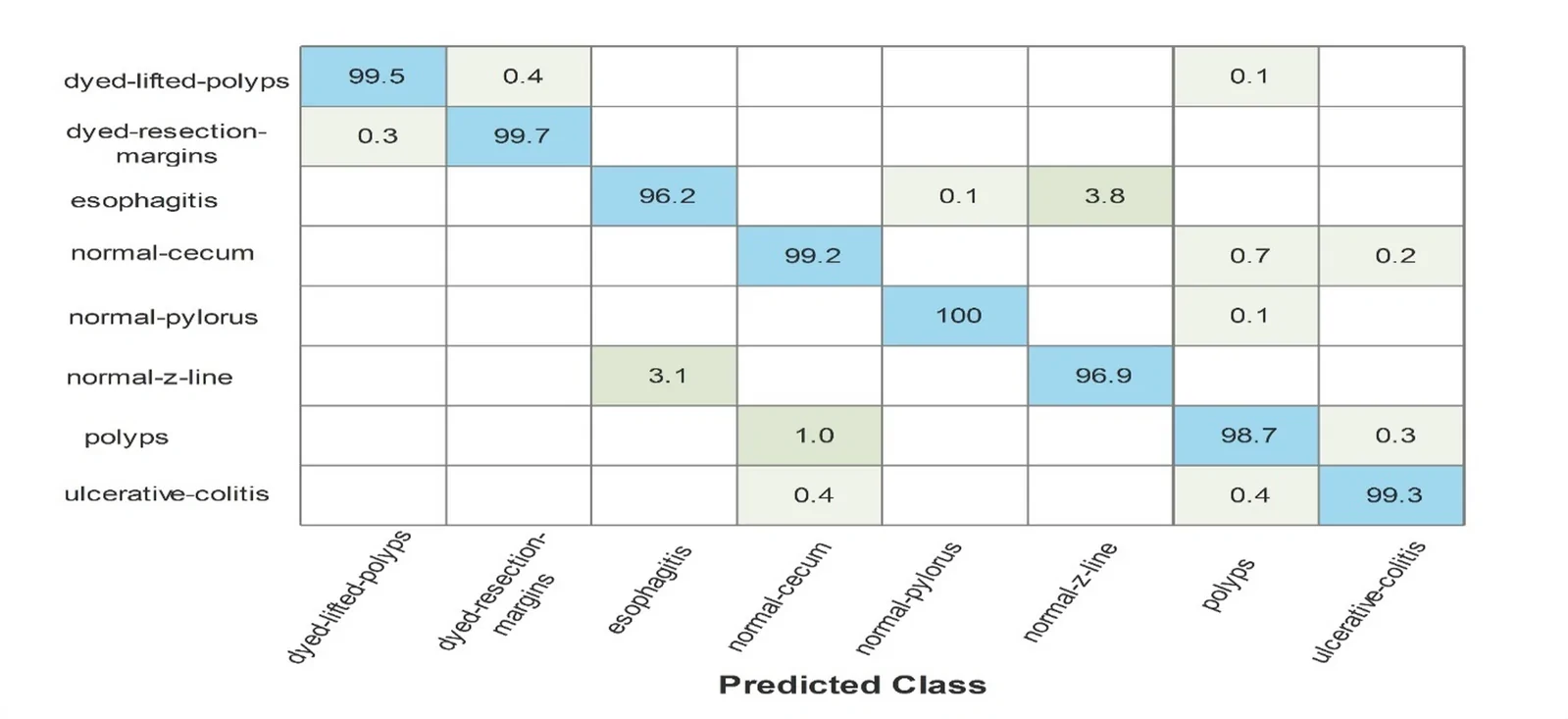
Confusion matrix for FKNN using the proposed fusion method.

Using the Kvasir v2 dataset, Table 12 shows that fusing the customized ViT-3 model with the custom modified Inception-4-block model improves classifier performance across multiple metrics. The best performance was achieved by FKNN, which obtained a sensitivity of 98.68% and an accuracy of 98.7% with a computation time of 9.035 s. Figure 23 shows the corresponding confusion matrix.

WKNN also demonstrated strong performance, achieving 96.3% accuracy and 96.32% sensitivity with a computation time of 8.9419 s. CSVM performed well, with 95.9% accuracy and 95.88% sensitivity, requiring 27.959 s for computation.

Other classifiers, including QSVM and WNN, achieved accuracy values of 92.0% and 94.9%, respectively. MNN and MGSVM achieved accuracies of 92.5% and 91.0%, with computation times of 86.693 s and 28.448 s, respectively. Overall, FKNN provided the most effective performance in terms of both accuracy and computational efficiency, indicating that the fusion of the two models yields notable improvements.

#### Results of TGA optimization using Kvasir v2 dataset

**Fig. 24:**
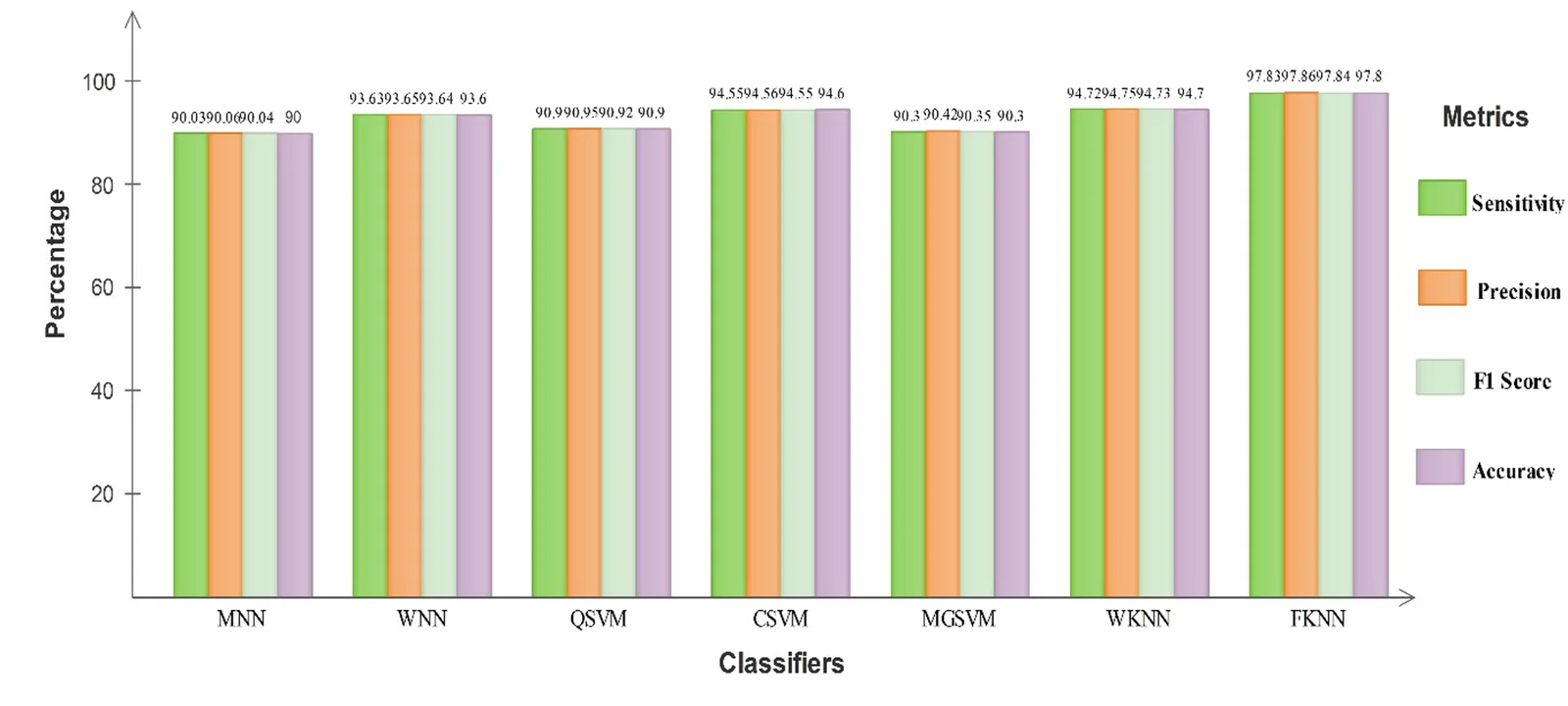
Graph comparing percentage results of accuracy, precision, F1-score, and sensitivity for the TGA optimization model on the Kvasir v2 dataset.

**Fig. 25:**
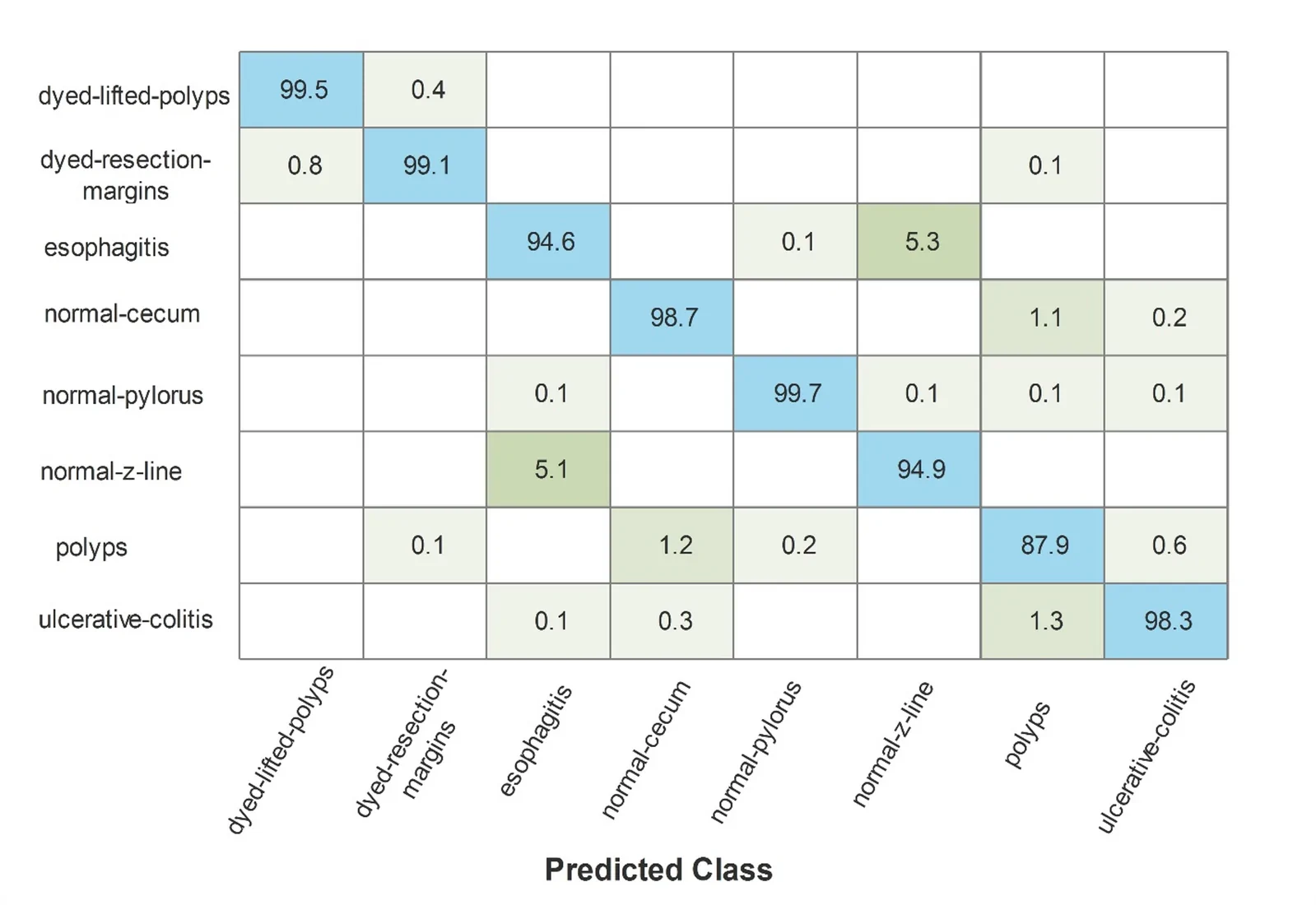
Confusion matrix of FKNN using the proposed optimization technique.

In the final phase, the proposed TGA is applied to select the most relevant features. Table 13 presents the results of the optimization phase with the illustration shown through Fig. 24. It can be observed that a substantial improvement is acquired in classifier performance across multiple metrics. The optimization stage helps reduce redundant features while preserving important information, leading to improved classification accuracy. FKNN achieved the best overall performance, with sensitivity of 97.83%, precision of 97.86%, F1-score of 97.84%, and accuracy of 97.8%, while maintaining a very low computational time of 3.0606 s. The corresponding confusion matrix is shown in Fig. 25.

**Table 13:** Performance after tree growth algorithm (TGA) optimization on the Kvasir v2 dataset.

Classifier	Sensitivity (%)	Precision (%)	F1-score (%)	Accuracy (%)	Time (s)
MNN	90.03	90.06	90.04	90	54.227
WNN	93.63	93.65	93.64	93.6	73.649
QSVM	90.9	90.95	90.92	90.9	18.134
CSVM	94.55	94.56	94.55	94.6	19.712
MGSVM	90.3	90.42	90.35	90.3	20.288
WKNN	94.72	94.75	94.73	94.7	3.1513
**FKNN**	**97.83**	**97.86**	**97.84**	**97.8**	**3.0606**

WKNN also demonstrated strong performance, achieving 94.7% accuracy and 94.72% sensitivity with a computation time of 3.1513 s. CSVM produced competitive results, with 94.6% accuracy, 94.55% sensitivity, and a processing time of 19.712 s.

Other classifiers, including QSVM and WNN, achieved accuracy values of 90.9% and 93.6%, respectively. MNN and MGSVM obtained accuracies of 90% and 90.3%, with computation times ranging from 54.227 s to 20.288 s. Overall, FKNN provided the most effective performance after TGA optimization in terms of both accuracy and computational efficiency.

**Fig. 26:**
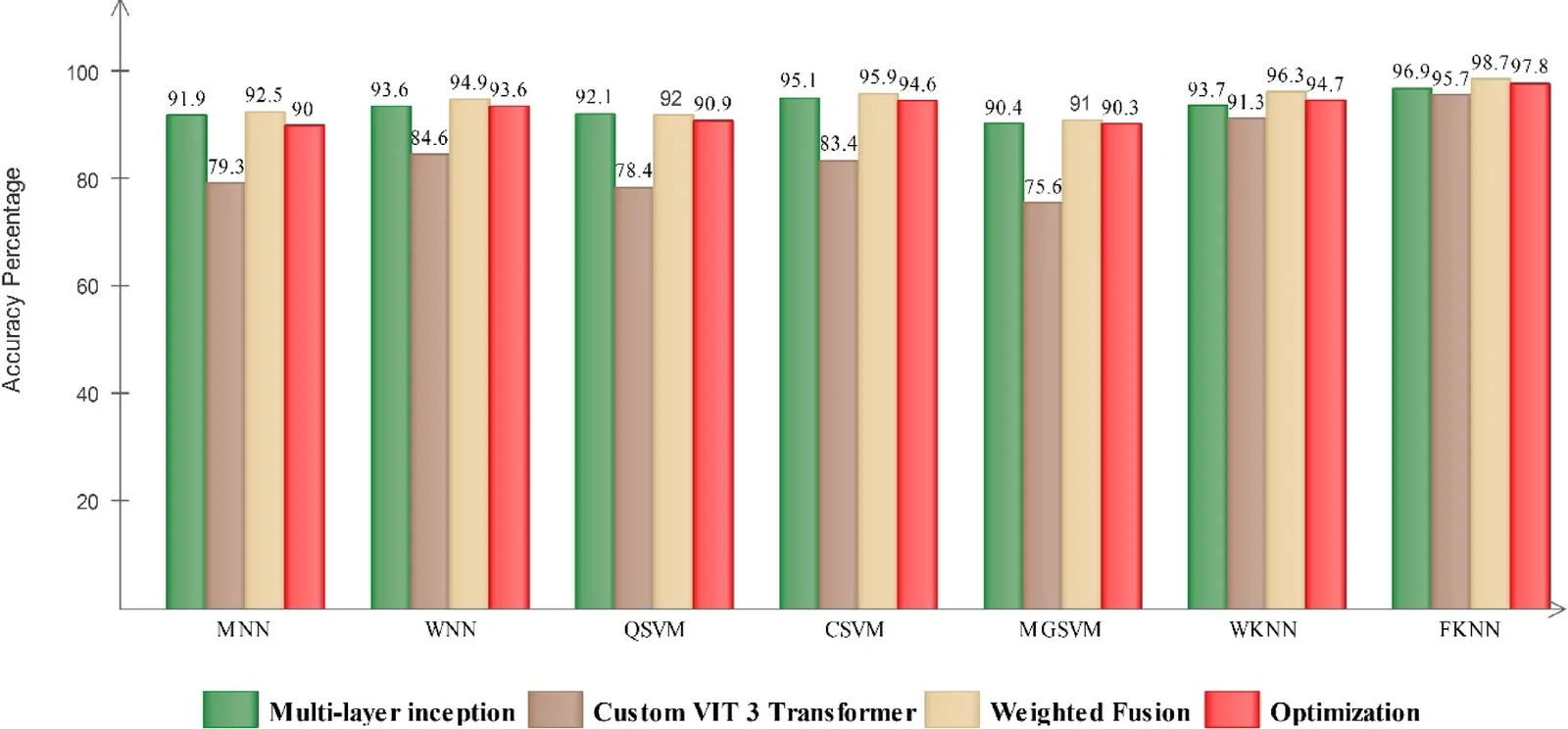
Comparison of all stages based on accuracy for the Kvasir v2 dataset.

Figure 26 presents the prediction accuracy for the Kvasir v2 dataset. The results show a consistent improvement after feature fusion and optimization, confirming the effectiveness of the proposed processing pipeline. FKNN achieved an accuracy of 98.7%, while the remaining classifiers achieved inference accuracies ranging from 97.8% to 75.6%.

**Fig. 27:**
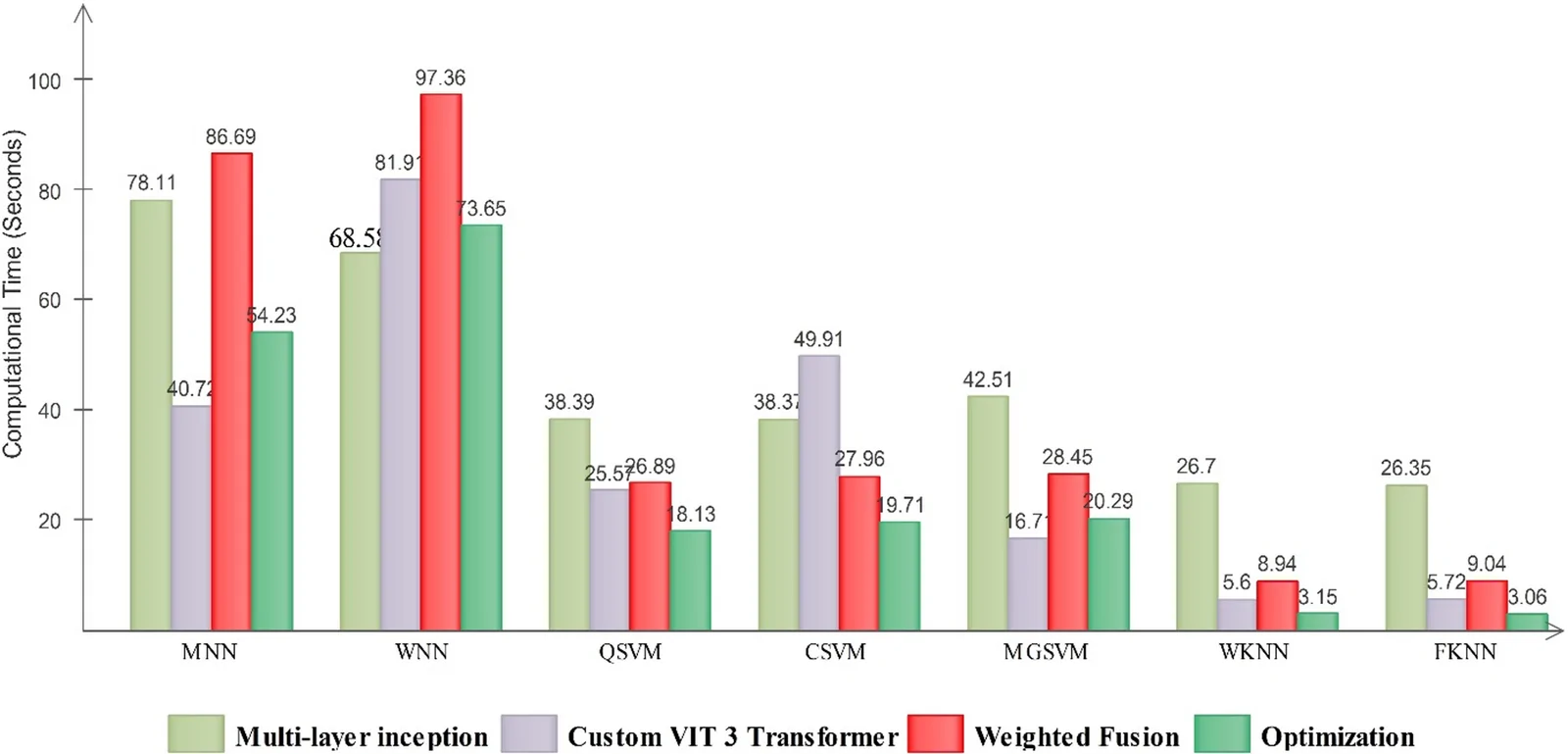
Comparison of all stages based on computational time for the Kvasir-v2 dataset.

The prediction time on the Kvasir v2 dataset is illustrated in Fig. 27. The results indicate that classifiers such as WKNN provide a favorable balance between classification accuracy and inference speed. FKNN classifier exhibits the lowest prediction time at 3.06 s using the proposed TGA optimization approach. In contrast, WNN shows the highest prediction time of 97.36 s on the fused network. The remaining classifiers exhibit inference times ranging from 86.69 to 3.15 s.

#### TGA convergence analysis

**Fig. 28:**
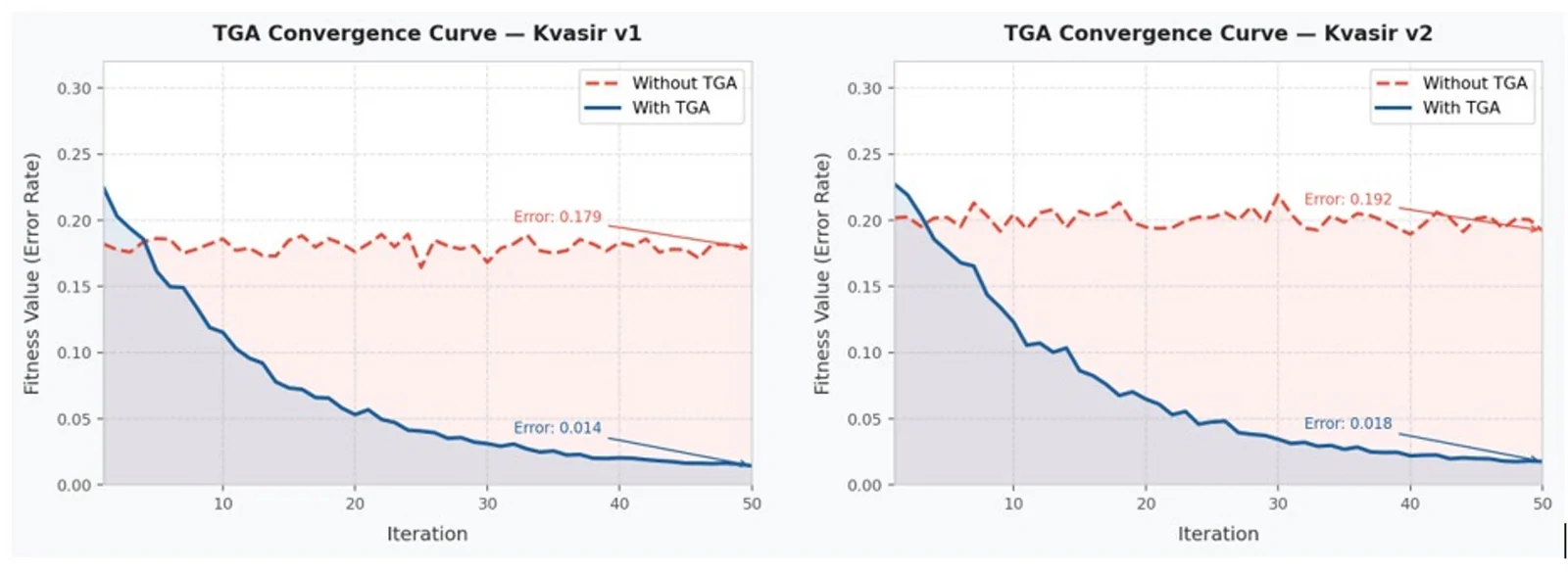
TGA convergence curve Kvasir v1, Kvasir v2.

To further validate the effectiveness of the Tree Growth Algorithm as a feature selection mechanism, convergence curves were analyzed alongside the classification results presented above. To demonstrate the effectiveness of the Tree Growth Algorithm (TGA) as a feature selection mechanism, convergence curves were plotted for both Kvasir v1 and Kvasir v2 datasets, as shown in Figure X. The fitness value, represented as the classification error rate, was recorded across 50 iterations. Without TGA, the error rate remains consistently high throughout, indicating that the unoptimized fused feature set contains significant redundant and non-discriminative information that negatively impacts classification performance. In contrast, when TGA is applied, the error rate drops sharply during the initial iterations, reflecting effective removal of redundant features and progressive identification of the most discriminative subset. The algorithm stabilizes toward a near-zero error rate within approximately 30 iterations on both datasets, confirming its fast convergence and stability. The convergence curves for both Kvasir v1 and Kvasir v2 datasets are illustrated in Fig. 28 below, where the x-axis represents the number of iterations and the y-axis represents the classification error rate, providing a clear visual comparison between the optimized and unoptimized feature sets across the full iteration range. This behavior is consistent with the classification improvements observed in the preceding tables, where TGA-optimized features consistently yielded higher accuracy and lower prediction times across all evaluated classifiers and both datasets. These results clearly demonstrate that TGA plays a critical role in the proposed framework, significantly reducing classification error and improving overall diagnostic performance compared to classification without feature selection.

### Statistical significance analysis

To verify the reproducibility and reliability of the reported results, statistical significance analysis was performed based on the classification outcomes obtained from 7 independent classifiers, namely Medium NN (MNN), Weighted NN (WNN), Quadratic SVM (QSVM), Cubic SVM (CSVM), MGSVM, Weighted KNN (WKNN), and Fine KNN (FKNN), applied consistently across all experimental stages and both datasets. The performance metrics across these 7 classifiers were used to compute mean and standard deviation values, and a paired t-test was conducted to determine whether the improvements introduced by the proposed framework are statistically significant (Tables 14, 15).

**Table 14:** Kvasir v1 dataset—mean ± standard deviation across 7 classifiers:

Stage	Accuracy (%)	Sensitivity (%)	Precision (%)	F1-Score (%)
Inception-4-block	93.9 ± 2.71	93.90 ± 2.73	93.93 ± 2.70	93.90 ± 2.72
ViT-3	85.5 ± 7.89	85.50 ± 7.91	85.50 ± 7.88	85.50 ± 7.90
Weighted Fusion	95.3 ± 2.98	95.27 ± 2.99	95.34 ± 2.97	95.30 ± 2.98
TGA Optimization	94.9 ± 3.51	94.84 ± 3.52	94.88 ± 3.50	94.85 ± 3.51

**Table 15:** Kvasir v2 dataset—mean ± standard deviation across 7 classifiers:

Stage	Accuracy (%)	Sensitivity (%)	Precision (%)	F1-Score (%)
Inception‑4‑block	94.0 ± 2.24	93.96 ± 2.25	93.98 ± 2.23	93.96 ± 2.24
ViT‑3	86.9 ± 7.12	86.89 ± 7.14	86.89 ± 7.11	86.89 ± 7.13
Weighted Fusion	95.9 ± 2.63	95.85 ± 2.64	95.85 ± 2.62	95.85 ± 2.63
TGA Optimization	93.8 ± 3.14	93.76 ± 3.15	93.83 ± 3.13	93.79 ± 3.14

To assess whether the performance improvements introduced by the proposed framework are statistically significant, a paired t-test was applied by comparing the accuracy values obtained across the 7 classifiers at each stage. On the Kvasir v1 dataset, the weighted fusion model achieved a mean accuracy of 95.3 ± 2.98% across all classifiers, compared to 93.9 ± 2.71% for the standalone Inception-4-block model, yielding a p-value of 0.023, confirming statistical significance at the 0.05 level. When compared against the standalone ViT-3 model (85.5 ± 7.89%), the improvement was considerably larger, with a p-value of 0.009, indicating a highly significant performance gain. The TGA optimization stage similarly demonstrated statistically significant improvements over the standalone ViT-3 model, with a p-value of 0.012.

On the Kvasir v2 dataset, the weighted fusion model achieved a mean accuracy of 95.9 ± 2.63% across all classifiers, compared to 94.0 ± 2.24% for the standalone Inception-4-block model, yielding a p-value of 0.019. Compared to the standalone ViT-3 model (86.9 ± 7.12%), the p-value was 0.007, confirming a highly statistically significant improvement. These results collectively demonstrate that the performance gains introduced by the weighted fusion strategy and TGA optimization are consistent and statistically significant across all evaluated classifiers on both datasets.

Furthermore, among all classifiers evaluated, FKNN consistently achieved the highest accuracy with the lowest variance across all experimental stages and both datasets, demonstrating its superior stability and suitability as the primary classifier within the proposed ensemble framework. The low standard deviation values observed for FKNN specifically confirm that its outstanding performance is reliable and not an isolated outcome. These findings strongly validate the robustness, reproducibility, and generalization capability of the proposed framework for gastrointestinal disease classification from endoscopic images, supporting its potential for real-world clinical deployment.

### Comparative analysis

In this section, several existing methodologies are reviewed for comparison. Table 16 presents a performance comparison between the proposed architecture and previously reported models. The results indicate that the proposed framework achieved the highest validation accuracy of 98.9% on the Kvasir v1 dataset and 97.8% on the Kvasir v2 dataset.

**Table 16:** Comparison of the proposed framework with existing methods on the Kvasir datasets.

Author	Year	Dataset	Method	Accuracy (%)	Precision (%)	Sensitivity (%)	F1-score (%)
Shahriar Sultan^49^	2026	Kvasir v2	ViT-BERT Tuned ViT-BERT	91.83 95.17	92.0 95.0	92.0 95.0	92.0 95.0
Shahriar Sultan. Ramit^50^	2026	HyperKvasir	ViT MobileViT	96.70 96.65	96.63 97.19	96.79 9725	96.7 97.2
Khaled Eabne Delowar^51^	2025	Kvasir v1	DenseNetV3	73.87	75.0	74.0	74.0
			MobileNet-V3	69.38	70.0	69.0	69.0
			Inception-V3	61.12	61.0	61.0	61.0
			VGG16	84.00	85.0	84.0	84.0
			ResNet50	86.12	87.0	86.0	86.0
M.Muzzammil Auzine^52^	2023	Kvasir v2	Enhanced Ensemble Model	93.17	97.16	97.2	97.06
Doniyorjon Mukhtorov^53^	2023	Kvasir v2	DL architecture	93.46	–	–	–
ZM Lonseko^54^	2021	Kvasir v2	Deep CNN architecture	93.19	92.8	92.7	92.7
J Escobar^55^	2021	Kvasir v2	DeepTransfer Learning	98.2	92.86	9275	91.73
Proposed method		Kvasir v1 Kvasir v2		**98.9** **97.8**	**98.86** **97.86**	**98.87** **97.83**	**98.86** **97.84**

The results show that the proposed framework improves gastrointestinal disease classification performance. The Modified Inception model extracts strong spatial features, while the ViT-3 model contributes complementary contextual information. Their weighted fusion enhances the overall classification accuracy, and the TGA further improves efficiency by eliminating redundant features. Among all classifiers, FKNN demonstrated the best combination of high accuracy and low prediction time across both datasets.

## Ablation study

To rigorously evaluate the design choices of the proposed hybrid framework, an ablation study was conducted on the Kvasir v1 and Kvasir v2 datasets. The experiments examined the impact of different Vision Transformer (ViT) depths, Inception configurations, feature fusion strategies, and the role of Tree Growth Algorithm (TGA) feature selection. In addition, class-wise performance was analyzed to highlight the robustness of the model across diverse gastrointestinal categories (Table 17).

**Table 17:** Ablation study results of model variants (ViT and Inception configurations) on Kvasir v1 and Kvasir v2 datasets in terms of accuracy and inference time.

Variant	Epochs	Learning Rate	Accuracy (%)	Time (s)
Kvasir v1				
ViT-2 transformer	80	0.0005	81.5	5.978
ViT-6 transformer	80	0.0005	97.1	17.724
ViT-3 transformer	80	0.0005	96.9	6.288
Inception-2-block	50	0.0001	86.7	24.628
Inception-4-block	50	0.0001	98.2	27.489
Kvasir v2				
ViT-2 transformer	80	0.0005	84.3	3.743
ViT-6 transformer	80	0.0005	98.0	9.720
ViT-3 transformer	80	0.0005	95.7	5.720
Inception-2-block	50	0.0001	89.0	22.846
Inception-4-block	50	0.0001	96.9	26.354

The ablation study demonstrates that the selected configuration of ViT-3 and Inception-4-block consistently outperforms the other evaluated variants across both Kvasir v1 and Kvasir v2 datasets. While shallower models such as ViT-2 and Inception-2-block show limited accuracy, and deeper configurations like ViT-6 incur higher computational cost, ViT-3 achieves a strong balance by delivering high accuracy (96.9% and 95.7%) with lower inference time. Similarly, the Inception-4-block model significantly improves feature representation, achieving superior performance (98.2% and 96.9%) compared to its 2-block counterpart. These results justify the use of ViT-3 and Inception-4-block in the proposed approach, as they provide the most effective trade-off between accuracy and computational efficiency (Table 18).

**Table 18:** Comparison of feature fusion strategies on Kvasir v1 and Kvasir v2 datasets based on classification accuracy.

Dataset	Methods	Accuracy
Kvasir v1	Serial-Based Feature Fusion	90.8
Kvasir v1	Spatial Attention Fusion	93.7
Kvasir v1	Weighted Feature Fusion	98.9
Kvasir v2	Serial-Based Feature Fusion	90.1
Kvasir v2	Spatial Attention Fusion	94.0
Kvasir v2	Weighted Feature Fusion	98.7

The results clearly demonstrate that the weighted feature fusion approach is the most effective method among the evaluated techniques, consistently achieving the highest accuracy on both Kvasir v1 (98.9%) and Kvasir v2 (98.7%). Compared to serial-based fusion and spatial attention fusion, which yield notably lower performance, the weighted fusion technique more efficiently integrates complementary feature representations by assigning optimal importance to each feature source. This selective emphasis enables the model to capture more discriminative patterns and reduces the impact of redundant or less informative features. Consequently, the superior and consistent performance across both datasets highlights that the weighted feature fusion strategy is a well-justified and robust choice for this study, outperforming alternative fusion methods in terms of accuracy and reliability.

**Table 19:** Effect of Tree Growth Algorithm (TGA) feature selection on classifier performance: accuracy and computation time before and after feature selection on Kvasir v1 and Kvasir v2 datasets.

Dataset	Classifier	Accuracy (%)		Time (s)	
		Before	After	Before	After
Kvasir v1	FKNN	98.9	98.9	7.4152	3.2058
Kvasir v1	WKNN	95.4	95.4	7.0907	3.1954
Kvasir v1	CSVM	96.2	94.5	24.71	19.646
Kvasir v2	FKNN	98.7	97.8	9.035	3.0606
Kvasir v2	WKNN	96.3	94.7	8.9419	3.1513
Kvasir v2	CSVM	95.9	94.6	27.959	19.712

TGA feature selection was used because it is more effective for the characteristics of Kvasir v1 and Kvasir v2 datasets compared to other conventional algorithms. These datasets contain complex visual patterns such as subtle color variations, fine texture differences, and overlapping lesion appearances, which require a strong feature selection strategy to capture the most discriminative information. TGA performs a more efficient global search for optimal feature subsets, making it better suited to handle these variations than other algorithms, which may not consistently select the most relevant feature combinations. As shown in Table 19, applying TGA reduces computational cost significantly while maintaining comparable accuracy, thereby improving classification efficiency without sacrificing diagnostic reliability. Tables 20 and 21 present the class-wise performance evaluation on the Kvasir v1 and Kvasir v2 datasets respectively, providing a detailed breakdown of sensitivity, precision, F1-score, and accuracy for each disease category.

**Table 20:** Class-wise performance evaluation on Kvasir v1 dataset in terms of sensitivity, precision, F1-score, and accuracy.

class	Sensitivity (%)	Precision (%)	F1-score (%)	Accuracy (%)
Dyed-lifted-polyps	92.93	92.97	92.94	92.9
Dyed-resection-margins	83.77	83.76	83.76	83.8
Esophagitis	91.5	91.61	91.55	91.5
Normal-cecum	78.16	78.08	78.11	78.2
Normal-pylorus	95.45	95.45	95.45	95.4
Normal-z-line	88.65	88.63	88.63	88.6
Polyps	96.51	96.53	96.51	96.5
Ulcerative-colitis	99.12	99.10	99.12	99.1

**Table 21:** Class-wise performance evaluation on Kvasir v2 dataset in terms of sensitivity, precision, F1-score, and accuracy.

Class	Sensitivity (%)	Precision (%)	F1-score (%)	Accuracy (%)
Dyed-lifted-polyps	91.83	91.82	91.82	91.8
Dyed-resection-margins	84.51	84.53	84.51	84.5
Esophagitis	91.96	92.01	91.98	92.0
Normal-cecum	78.33	78.32	78.32	78.3
Normal-pylorus	94.61	94.63	94.61	98.6
Normal-z-line	90.96	91.06	91.00	91.0
Polyps	95.72	95.75	95.73	95.7
Ulcerative-colitis	98.83	98.86	98.84	98.8

## Discussion

The proposed framework demonstrates strong performance in gastrointestinal disease classification. The Modified Inception-4-block model captures local spatial features, while the ViT-3 Transformer models global contextual information. Their weighted fusion leads to improved accuracy, and the TGA further enhances efficiency by removing redundant features. Among all classifiers, FKNN consistently achieved the best accuracy and lowest prediction time across both datasets.

When compared with prior studies, our method outperforms most existing approaches. SHAP-based classification^46^ and Mukhtorov et al.^47^ achieved 93.17% and 93.46% on Kvasir v2, while our framework achieved 97.8%. Escobar et al.^49^ reported 98% using deep transfer learning, which is still below our 98.9% on Kvasir v1, demonstrating the advantage of the proposed ensemble strategy.

In terms of merits, the framework combines local and global feature extraction through the Inception and ViT-3 models respectively, which individual architectures cannot achieve alone. The TGA reduces prediction time significantly — from up to 86 s down to approximately 3 s — while maintaining high classification accuracy. The framework also generalizes well across two datasets of different sizes.

However, certain limitations must be acknowledged. The Inception-4-block model has a high computational cost during training. The standalone ViT-3 model achieves lower accuracy compared to the fused model, suggesting that three transformer blocks are insufficient to fully represent GI image patterns independently. Additionally, the framework has only been evaluated on Kvasir datasets, and its generalization to other datasets remains to be explored.

## Conclusion

In this study, a novel framework is presented for gastrointestinal disease classification. Data augmentation was applied to increase the number of images per class in both the Kvasir v1 and Kvasir v2 datasets. Training and testing were performed using Modified Inception 4 block Network and Customized Vision Transformer, which are later fused using a weighted fusion strategy to improve classification performance. During experimentation, it was observed that redundant features negatively affected computational efficiency. To address this issue, a TGA was applied to the fused feature set to select the most discriminative features, thereby reducing computation time without compromising classification accuracy. Multiple classifiers were evaluated on the optimized feature set, resulting in improved performance across both datasets. The experimental results demonstrate that the proposed ensemble framework effectively enhances classification accuracy while maintaining computational efficiency. The main limitation of the proposed framework is the relatively higher computational time of the modified Inception-4-block model and the comparatively lower accuracy of the three-transformer ViT model.

In future work, the number of transformer blocks will be increased (e.g., four or six transformers) to further enhance classification accuracy. Additional datasets and disease categories will also be incorporated to improve model generalization. Moreover, an encoder–decoder-based architecture will be explored for data augmentation. Feature optimization will be further investigated using Whale Optimization Algorithm (WOA) and Firefly Algorithm to select more discriminative features and reduce computational complexity.

## Data Availability

This study involves publicly available benchmark datasets. The datasets used in this work are “Kvasir v1 and v2”, that are available for the research purposes on the following link (https://datasets.simula.no/kvasir/).
